# Reaction of *N*-Acetylcysteine with Cu^2+^: Appearance of Intermediates with High Free Radical Scavenging Activity: Implications for Anti-/Pro-Oxidant Properties of Thiols

**DOI:** 10.3390/ijms23116199

**Published:** 2022-05-31

**Authors:** Ivan Valent, Lucie Bednárová, Igor Schreiber, Juraj Bujdák, Katarína Valachová, Ladislav Šoltés

**Affiliations:** 1 Department of Physical and Theoretical Chemistry, Faculty of Natural Sciences, Comenius University, Mlynská dolina, Ilkovičova 6, 842 15 Bratislava, Slovakia; juraj.bujdak@uniba.sk; 2 Institute of Organic Chemistry and Biochemistry of the Czech Academy of Sciences, Flemingovo náměstí 542/2, 160 00 Praha 6, Czech Republic; lucie.bednarova@uochb.cas.cz; 3 Department of Chemical Engineering, Faculty of Chemical Engineering, University of Chemistry and Technology, Prague, Technická 5, 166 28 Praha 6, Czech Republic; igor.schreiber@vscht.cz; 4 Global Change Research Institute, Czech Academy of Sciences, Bělidla 986/4a, 603 00 Brno, Czech Republic; 5 Centre of Experimental Medicine of Slovak Academy of Sciences, Dúbravská cesta 9, 841 04 Bratislava, Slovakia; katarina.valachova@savba.sk (K.V.); ladislav.soltes@savba.sk (L.Š.)

**Keywords:** autoxidation, copper catalysis, circular dichroism, exciton coupling, disulfide, dicopper complex, dioxygen activation

## Abstract

We studied the kinetics of the reaction of *N*-acetyl-l-cysteine (NAC or RSH) with cupric ions at an equimolar ratio of the reactants in aqueous acid solution (pH 1.4–2) using UV/Vis absorption and circular dichroism (CD) spectroscopies. Cu^2+^ showed a strong catalytic effect on the 2,2′-azino-bis(3-ethylbenzothiazoline-6-sulfonate) radical (ABTSr) consumption and autoxidation of NAC. Difference spectra revealed the formation of intermediates with absorption maxima at 233 and 302 nm (*ε*_302_/Cu > 8 × 10^3^ M^−1^ cm^−1^) and two positive Cotton effects centered at 284 and 302 nm. These intermediates accumulate during the first, O_2_-independent, phase of the NAC autoxidation. The autocatalytic production of another chiral intermediate, characterized by two positive Cotton effects at 280 and 333 nm and an intense negative one at 305 nm, was observed in the second reaction phase. The intermediates are rapidly oxidized by added ABTSr; otherwise, they are stable for hours in the reaction solution, undergoing a slow pH- and O_2_-dependent photosensitive decay. The kinetic and spectral data are consistent with proposed structures of the intermediates as disulfide-bridged dicopper(I) complexes of types *cis*-/*trans*-Cu^I^_2_(RS)_2_(RSSR) and Cu^I^_2_(RSSR)_2_. The electronic transitions observed in the UV/Vis and CD spectra are tentatively attributed to Cu(I) → disulfide charge transfer with an interaction of the transition dipole moments (exciton coupling). The catalytic activity of the intermediates as potential O_2_ activators via Cu(II) peroxo-complexes is discussed. A mechanism for autocatalytic oxidation of Cu(I)–thiolates promoted by a growing electronically coupled –[Cu^I^_2_(RSSR)]*_n_*– polymer is suggested. The obtained results are in line with other reported observations regarding copper-catalyzed autoxidation of thiols and provide new insight into these complicated, not yet fully understood systems. The proposed hypotheses point to the importance of the Cu(I)–disulfide interaction, which may have a profound impact on biological systems.

## 1. Introduction

Thiol compounds are frequently used as drugs with a variety of therapeutic effects. Thiol functional group(s) containing drugs can reduce free radicals, regulate cellular redox state, and form stable complexes with heavy metals [1]. Although the most common thiol-derived drugs are represented by relatively simple molecules, which have been in use for decades, the exploration of their properties and mechanisms of therapeutic action continues. Such research aims to optimize clinical applications and provides new perspectives on the development of more effective treatments.

One of the well-established thiol drugs, *N*-acetyl-l-cysteine (NAC), is known as an antioxidant and glutathione (GSH) precursor. NAC has been used in clinical practice for a long time as a mucolytic agent and for the treatment of numerous disorders such as various intoxications (including heavy metal poisoning), ischemia-reperfusion cardiac injury, acute respiratory distress syndrome, bronchitis, HIV/AIDS, and psychiatric disorders [2,3]. In addition, the anticancer activity of NAC in combination with copper ions has been reported [4], motivating current research of NAC as a potential anticancer agent [5,6,7].

The molecular mechanisms underlying the biological activity of NAC are only partially understood [8]. The mucolytic effect is ascribed to the efficient disulfide breaking potential of NAC [9], while the antidotal effects against heavy metal intoxications can be explained by the capability of NAC to chelate various metal ions [2]. The antioxidant activity of NAC is most probably due to its rapid reactions with free radicals such as ^•^OH, ^•^NO_2_, CO_3_^•−^, and thiyl radicals [2,9]. Yet, direct reactions of NAC with O_2_^•−^, H_2_O_2_, and peroxynitrite are relatively slow [2,10]. An indirect antioxidant effect of NAC is attributed to its functioning as a cysteine precursor in the biosynthesis of the key cellular antioxidant GSH [2,9]. The conventional view of the mechanisms of NAC action has recently been put in question as hydrogen sulfide and sulfanes produced from NAC might account for the antioxidative and cytoprotective activities that have previously been ascribed to NAC or NAC-derived glutathione [8,11].

On the other hand, pro-oxidant behavior of NAC has also been demonstrated [4,12,13,14,15]. It is generally accepted that pro-oxidant activity of thiols originates from reactive oxygen species (ROS) such as O_2_^•−^, H_2_O_2_, and ^•^OH generated by autoxidation (i.e., reaction with atmospheric oxygen) of thiols in the presence of redox-active transition metals, mainly Cu(I/II) and Fe(II/III) [2,15,16,17,18,19]. However, ROS-independent apoptosis of cancer cells enhanced by NAC has also been reported [20,21,22]. Recently, a combination of NAC and copper has been suggested as a potential antiviral treatment against SARS-CoV-2 [23].

Although the chemistry of thiols and particularly their interactions with copper ions have been studied for more than a century [18,24,25,26,27,28,29,30,31,32,33,34,35,36,37,38,39,40,41,42,43,44,45,46], still little agreement exists on the fundamental mechanisms of the chemical processes involved. For instance, the classical problem of copper-catalyzed autoxidation of cysteine has remained a controversial issue as to the nature of catalytic species and the oxidation state of copper in such catalysts [29,35,36,40,42,47]. Moreover, the substantial production of ROS as a result of the thiol–copper interaction in the presence of dioxygen [16,18] is questioned by some authors [48]. The original presumptions that hydroxyl radicals formed by a Fenton-like reaction of H_2_O_2_ with Cu^+^ [17] cause irreversible damage of biomacromolecules were excluded, for example, by Macomber et al. [49]. The reasons for such criticism include thermodynamic disfavor of production of high-energy radical species [34], possible direct scavenging of the formed ROS in situ by the excess of thiol [49], and prevention of the Fenton reaction by rapid chelation of Cu(I) by thiols [19,50]. Some authors argue that the production of ^•^OH is extremely slow in Cu(I)/Cu(II)/H_2_O_2_/O_2_ systems at pH 8 and suggest Cu(III) species as a powerful oxidant [51].

The aim of this study was to contribute to further understanding of the complex processes occurring during the copper-catalyzed oxidation of thiols. We focused on the behavior of NAC in the presence of copper ions under acidic conditions, where the reaction is slow enough to be followed by the conventional spectroscopic methods—UV/Vis absorption and circular dichroism (CD). The radical cation (ABTSr) derived from 2,2′-azino-bis(3-ethylbenzothiazoline-6-sulfonate) was used to monitor the kinetics of the initial phase of the reaction and to search for electron-donating species produced during the reaction. The results reveal the formation of several intermediates, which probably play important roles in the mechanism of thiol–copper interaction and autoxidation of thiols from both the chemical and biological points of view.

## 2. Results

### 2.1. Kinetics of ABTSr Scavenging: Cu^2+^ Catalysis and H^+^ Inhibition

Our previous investigations [52] have shown that ABTSr is a suitable substrate to study the initial stages of the Cu(II)–thiol interaction. As a single-electron acceptor, ABTSr effectively competes with O_2_ for Cu(I), while its direct reaction with (RSH) is relatively slow. Thus, further reactions of Cu(I) are suppressed until the ABTSr is consumed and simplified initial kinetics can be followed. Additionally, ABTSr is a good indicator of the oxidation state of copper when added during the reaction (see Section 2.4).

**Figure 1:**
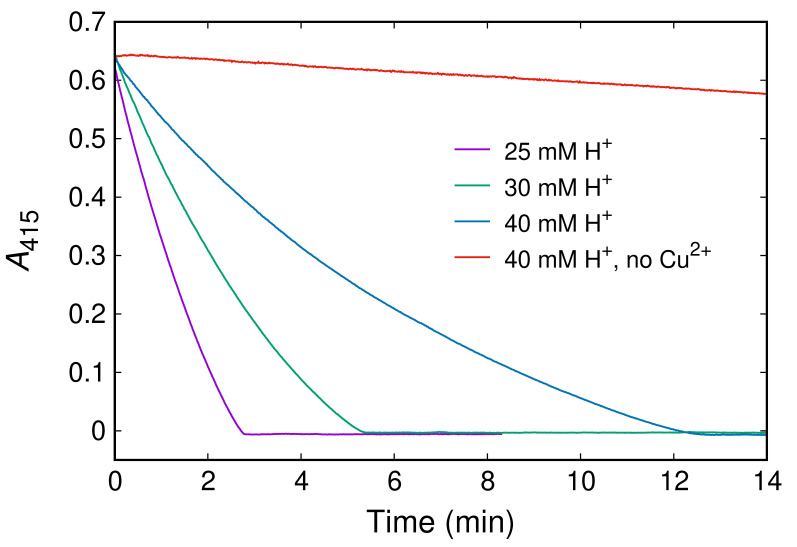
Kinetics of ABTSr decay at 415 nm during the reaction with NAC in the presence of Cu^2+^ at [H^+^] = 25, 30, and 40 mM. Other initial concentrations (in order of addition): 300 μM Cu^2+^, 20 μM ABTSr, 300 μM NAC. The records were acquired on the diode array spectrophotometer with an open (off) shutter at 20 °C.

As described [52], some thiols, e.g., bucillamine, show zero-order kinetics in ABTSr consumption under appropriate conditions, instead of the more typical first-order kinetics. Moreover, in the case of NAC, reaction conditions can be found, where simultaneous zero- and first-order kinetics in ABTSr decay are observed (Figure 1). Such observations are highly motivating, as the zero-order kinetics could be interpreted assuming the formation of an intermediate in a rate-determining step preceded its rapid reaction with ABTSr.

### 2.2. UV/Vis Absorption Spectra Reveal a Transient Characterized by an Absorption Maximum at 302 nm

**Figure 2:**
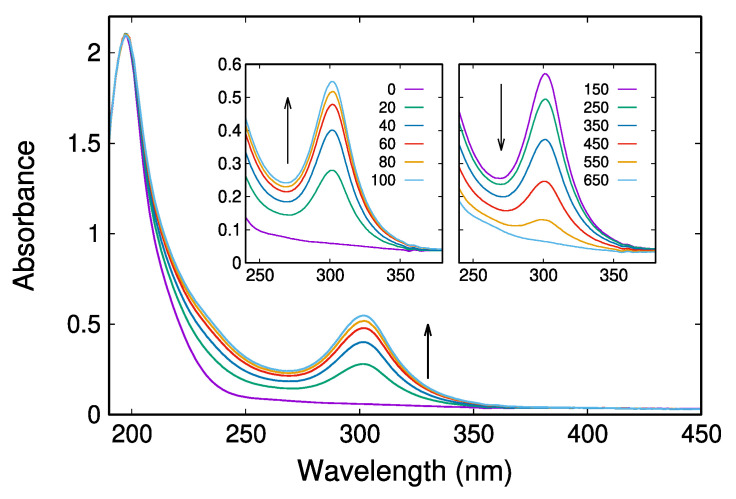
UV absorption spectra of a reaction solution initially containing 300 μM Cu^2+^ and 300 μM NAC in 40 mM HClO_4_ recorded at the given times in minutes (insets) from the beginning of the reaction. The records were acquired on the diode array spectrophotometer with a scan period of 20 s (shutter on) at 20 °C.

To search for tentative intermediates, the reaction solutions at various times in the absence and the presence of ABTSr were examined by UV/Vis and CD-spectroscopies. Indeed, the formation of a transient showing an absorption maximum at 302 nm was observed (Figure 2). The concentration of transient culminates usually after 80–140 min from the beginning of the reaction depending on conditions, and then slowly decays within ~11 h.

**Figure 3:**
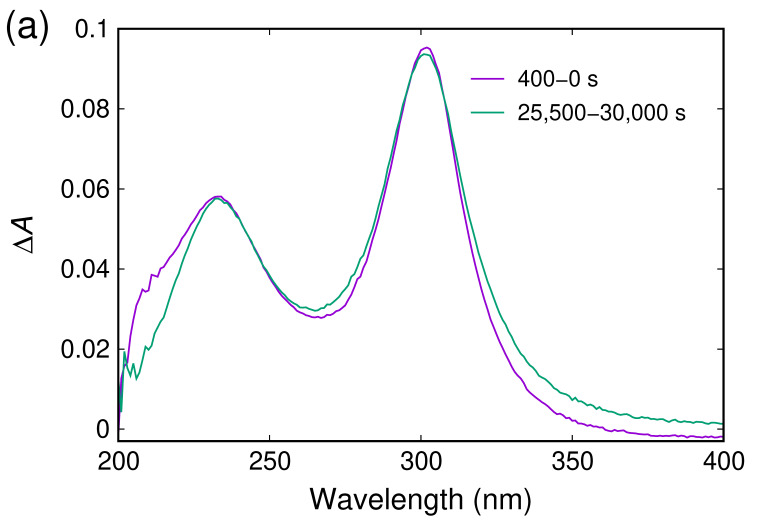

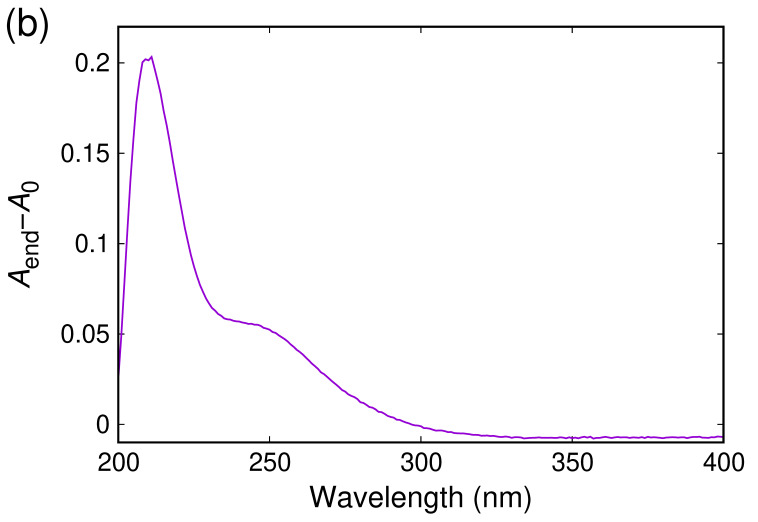
Difference absorption spectra from the experiment in Figure 2. The values of the absorbance difference between the given times (**a**) and between the end (>16 h) and the beginning of the reaction (**b**) are shown.

Difference absorption spectra, calculated as the subtraction of various time-scans, revealed, besides the maximum at 302 nm, a less intense maximum at 233 nm (Figure 3a). Although the intensity ratio of both maxima (*I*_233nm_/*I*_302nm_) seems to be conserved throughout the reaction progress, it is not evident whether they belong to the same intermediate. On the other hand, a difference spectrum obtained by subtracting the scan at the beginning (*t* = 0) from the scan at the end (*t* > 16 h) of the recording (Figure 3b) exhibits a peak at 210 nm and a shoulder at 240–250 nm, the latter typical for disulfides. Comparison of the difference spectrum in Figure 3b with a reference spectrum of an aqueous solution of pure diNAC confirms the well-known fact that the main product of a thiol autoxidation is the corresponding disulfide. However, a rough estimate indicates a substoichiometric conversion of NAC to diNAC, suggesting that a fraction of NAC remained unreacted, complexed with copper (as thiolate and/or disulfide), or converted to side products, e.g., sulfonic acid as supposed by Kachur et al. [18].

**Figure 4:**
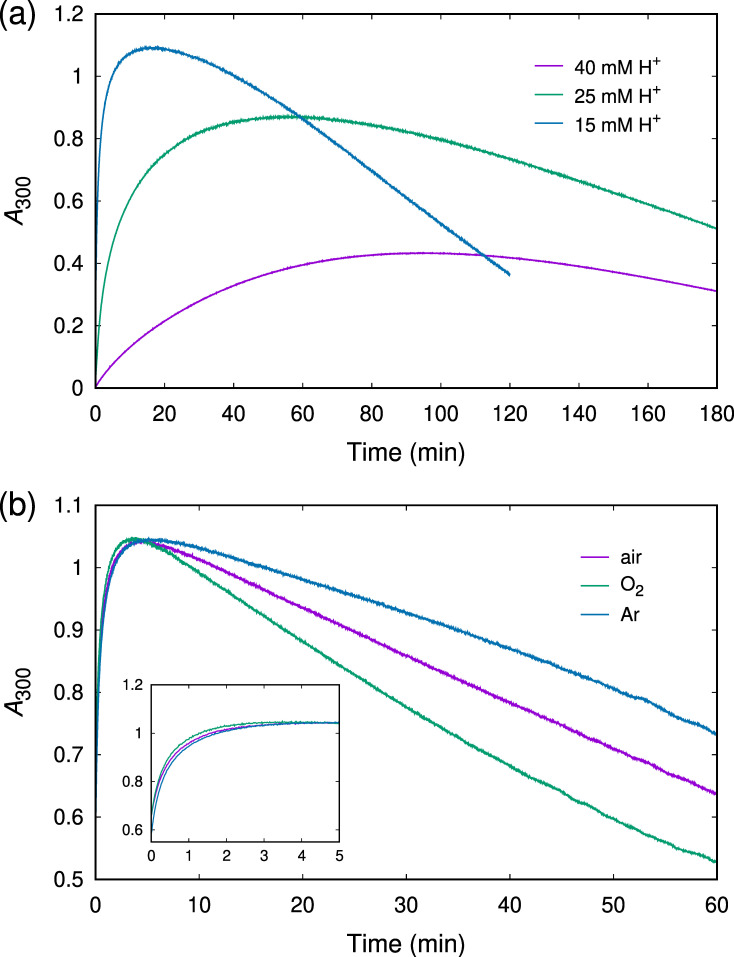
Time dependence of the absorbance at 300 nm of Cu–NAC mixtures (**a**) in various solutions of HClO_4_ and (**b**) in a 10 mM HClO_4_ solution (air) compared with solutions bubbled for 5 min by oxygen and argon before the reaction started. Initial concentrations: 300 μM Cu^2+^, 300 μM NAC. The records were acquired on the diode array spectrophotometer (shutter off) at 20 °C.

As in the kinetics of ABTSr decay (Figure 1), both the rate of production and decline of the 302 nm transient during the autoxidation is strongly pH-dependent (Figure 4a). Dioxygen has a negligible effect on the rate of production of the 302 nm transient (Figure 4b); however, the rate of its decay is significantly accelerated in an oxygenated solution compared to the reaction under the argon atmosphere. In addition, photosensitivity probably affects the rate of the *A*_300_-signal decrease (Figure S1 in Supplementary Materials). Additionally, it is not excluded that the circularly polarized light could influence the system behavior [53].

### 2.3. CD Spectroscopy Reveals Another Transient Showing Sigmoidal Production Kinetics

**Figure 5:**
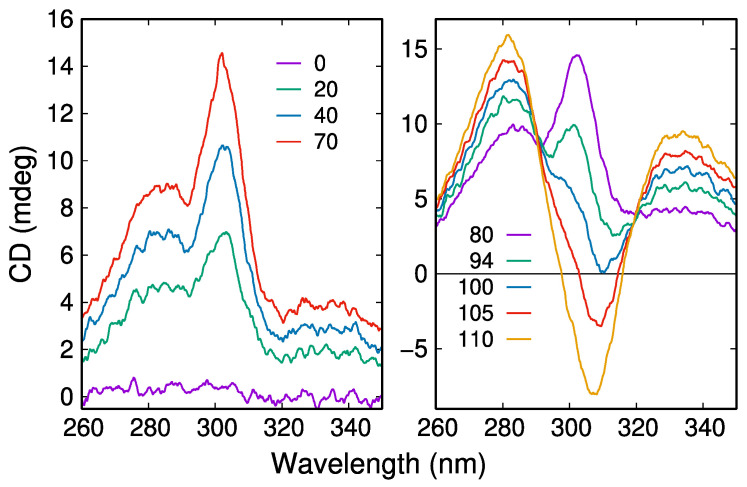
CD spectra of a Cu–NAC mixture at selected times (labels in minutes) from the start of the reaction. Initial concentrations: 40 mM HClO_4_, 300 μM Cu^2+^, 300 μM NAC; 12 °C.

The appearance of intermediates can also be followed using CD spectroscopy. Figure 5 shows the time evolution of CD spectra of the Cu–NAC reaction solution under the conditions of slow kinetics (40 mM HClO_4_, 12 °C). Two positive maxima at 284 and 302 nm progressively develop within 80 min from the beginning of the reaction (Figure 5, left).

After the 80 min period, dramatic changes in the CD spectrum are observed. The maximum at 284 nm slightly blue shifts and increases in intensity. The positive peak at 302 nm is gradually replaced by a negative one at ~308 nm. Following the sigmoidal course, it culminates at −150 mdeg after 5 h from the beginning of the reaction (not shown) and then very slowly (in >7 h) returns to zero. Simultaneously, a positive band forms centered at ~330 nm. We relate the CD spectral changes observed mainly after 80 min to the appearance of another transient compound.

**Figure 6:**
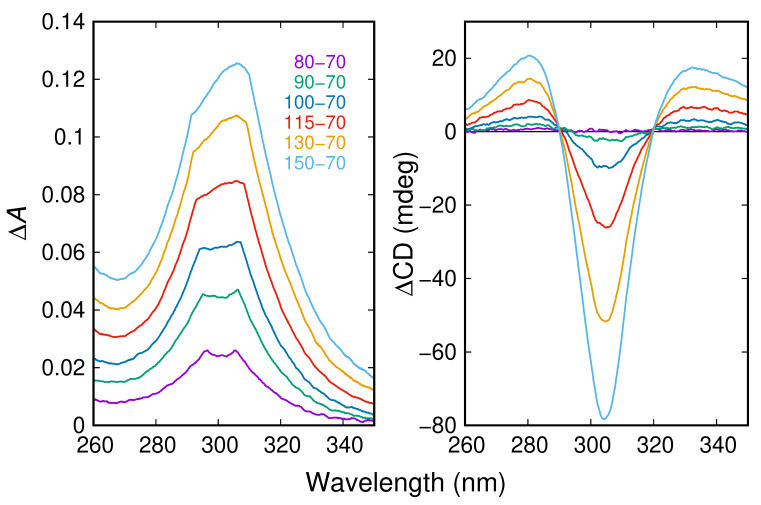
The difference absorption (**left**) and CD (**right**) spectra 80–150 min from the start of the reaction from the experiment in Figure 5. The spectrum at 70 min was taken as a reference. Time labels in minutes apply for both panels.

Further valuable information can be obtained from a detailed analysis of the difference spectra. The unusual behavior of the system is documented in Figure 6, which illustrates the period of 80–150 min with the maximal production rate of the second transient.

The differential absorbance (Figure 6, left) reveals a substantial deformation of the 302 nm maximum, whereas the differential ellipticity (Figure 6, right) demonstrates an almost linear increase in a uniform pattern. We explain the observed perturbation of the 302 nm absorption band by a coincidence of at least two processes. As the absorbance around 302 nm increases during the inspected period, it can be assumed that the first transient/intermediate is still produced; however, another species with similar spectral properties, possibly an isomer of the first intermediate or a compound containing the same chromophore, is consumed and thereby negatively contributes to the observed pattern. In addition, a contribution of the producing second transient to the multicomponent difference spectra should be considered.

The uniformity of the observed difference CD spectrum (Figure 6, right) suggests that it can be attributed to the second transient/intermediate as a species with the most dominant CD signal at this phase of the reaction. Interestingly, the rotational strength of the positive and negative Cotton effects, estimated by integration of the difference CD spectrum in the wavelength region of 250–445 nm, is almost equal (Figure S5c). This fact provides a possible interpretation of the obtained CD pattern as a spectrum arising from an exciton coupling [54]. Such an assumption is also consistent with a relatively high intensity of the CD signal. In this respect, the band split apparent in the CD spectrum (Figure 5, left), related to the first intermediate (or two isomers of it), could be explained analogously. As both positive and negative CD patterns in Figure 5 are centered around 300 nm, as is the lower energy absorption band in Figure 3a, it is reasonable to assume that all the observed transitions in this region are due to the same chromophore. An interaction of transition dipole moments of two or more such chromophores present in the same molecule could explain the observed spectroscopic features. Moreover, as discussed below, such considerations provide useful hints for structural proposals of the postulated intermediates.

**Figure 7:**
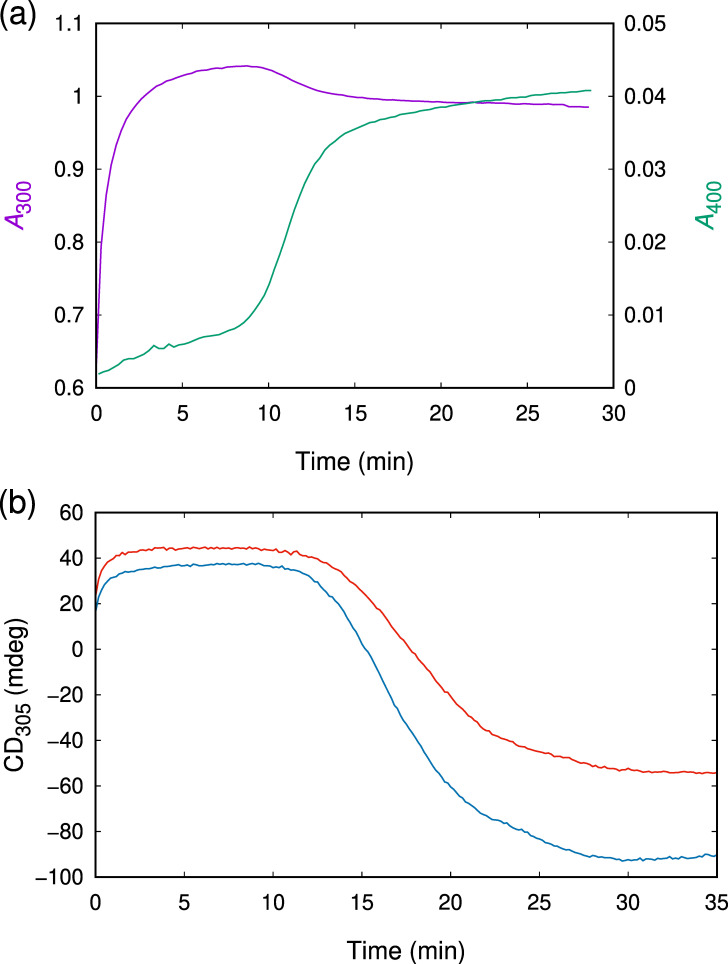
Time courses of the absorbances for 300 (magenta line) and 400 nm (green line) of a single measurement (**a**) and CD time records at 305 nm of two (red and blue lines) independent experiments (**b**). Initial concentrations: 10 mM HClO_4_, 300 μM NAC, 300 μM Cu^2+^; 20 °C. The records were acquired on the conventional double-beam spectrophotometer (**a**) with a period of 17 s alternating between 300 nm and 400 nm and on the CD spectrometer (**b**) with a data pitch of 10 s (shutter on).

The second intermediate manifests a broad positive band in the CD spectrum spanning the wavelength region of 320–445 nm. As the absorption spectrum also displays increasing activity in this region, when the second transient is formed, it is likely that such an increase in absorbance is due to the second intermediate. The first intermediate does not contribute to the light absorption above 400 nm (Figure 3a), so this wavelength can be selected to follow the production of the second transient by the standard UV/Vis absorption spectroscopy. Figure 7 shows time courses of the absorbances at 300 and 400 nm, recorded on a double-beam device switching between these two wavelengths, compared with another two experiments monitoring the CD signal at a fixed wavelength of 305 nm under the same conditions as the spectrophotometric measurement. Although the kinetics are significantly faster at a lower acidity and higher temperature, the sigmoidal (S-shaped) production curve for the second intermediate is clearly resolved.

Figure 7a confirms that a portion of the first intermediate (IM1) is consumed during the production period of the second intermediate (IM2), as indicated by the difference absorption spectra in Figure 6. Thus, it seems reasonable to assume that a certain amount of IM1 converts to IM2 during the reaction. The sigmoidal course of the IM2 production could then result from the consecutive steps: → IM1 → IM2. In such a case, a maximal rate of the IM2 production should coincide with a maximal concentration of IM1. However, Figure 7a,b demonstrate that both *A*_300_ and CD_305_ signals culminate before the substantial production of IM2 started. Conversely, the time when the maximal rate in the *A*_300_ decrease was attained is close to the time for the maximal rate in the *A*_400_ increase, as seen from the comparison of the inflection times in Figure 7a. This evidence supports the possible conversion of IM1 to IM2, but suggests that the sigmoidal production of IM2 (and also the sigmoidal consumption of some IM1) cannot originate from a simple consecutive reaction. We incline to a notion that both IM1 and IM2 are involved in an autocatalytic process, as discussed later. It should be noted here that, as Figure 7b illustrates, the sigmoidal course of the CD_305_ signal is not ideally reproducible as to the exact value of inflection time and the amounts of the produced IM1 and IM2. Such behavior is consistent with the assumption that the described complex process is governed by nonlinear kinetics, which can lead to sensitivity to the initial conditions.

### 2.4. The Observed Intermediates React Rapidly with ABTSr

**Figure 8:**
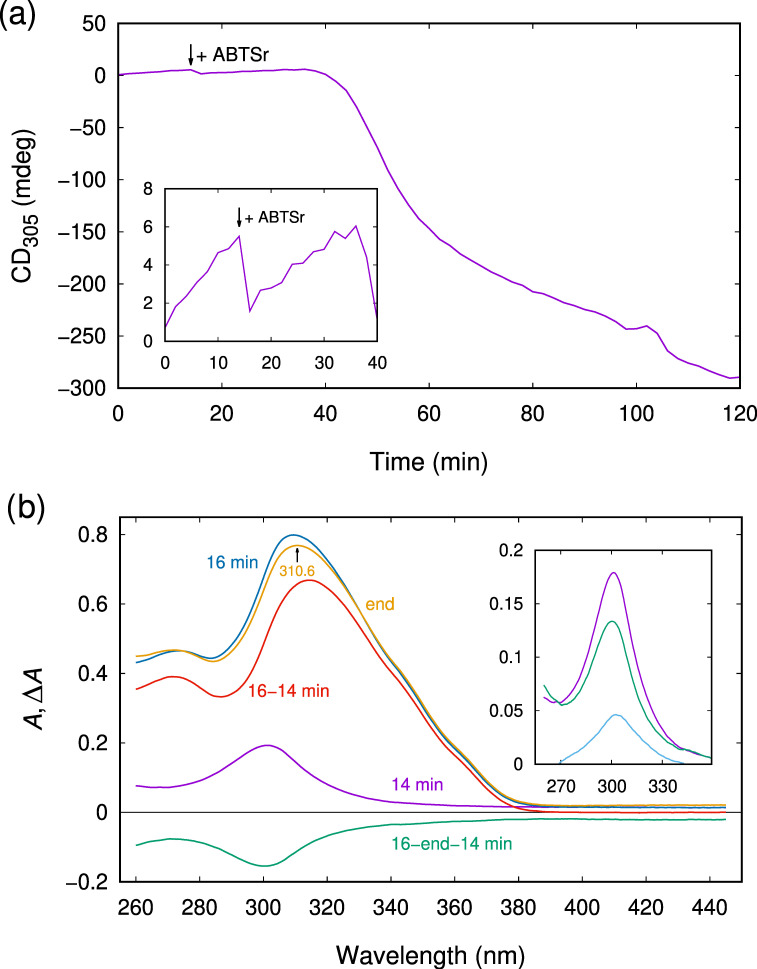
CD and UV/Vis spectrophotometric analysis of the effect of ABTSr addition (at 14 min) during the reaction of 300 μM Cu^2+^ with 300 μM NAC in 40 mM HClO_4_ at 20 °C. The spectra (445 → 260 nm, resolution 0.1 nm) are scanned in 2 min cycles at a rate of 100 nm min^−1^. (**a**) A time record of the ellipticity at 305 nm; 20 μM of ABTSr is added at 14 min (arrow) from the beginning of the reaction. (**b**) UV/Vis spectra plotted as absorbance (*A*) and difference absorbance (Δ*A*) vs. wavelength. Details are explained in the text.

To gain more information on the properties of the putative intermediates, a series of experiments with ABTSr additions was performed. If ~20 µM of the ABTS radical is initially present in the reaction solution under the conditions as in Figure 1 (40 mM HClO_4_), the CD spectrum attributed to the first intermediate does not appear until the ABTSr is completely consumed. If the ABTSr (20 µM) is added during the period of production of the IM1 (Figure 8), the blue color of ABTSr immediately disappears, and no absorption characteristic for the radical form is observed in the spectra.

The ABTSr addition causes a decrease in the CD_305_ signal by about 71% (shown magnified in the inset of Figure 8a) and then the signal increases with the continuing production of IM1. The decrease in the CD_305_ ellipticity can be attributed mainly to the consumption of IM1, as the added ABTSr shows only a minor contribution (Figure S2). In addition, as shown below, ABTSr rapidly converts to its reduced form (ABTS) upon addition to the reaction mixture, so that no ABTSr is detected in the subsequent spectral scan. The contribution of ABTS to the ellipticity in the studied spectral region is negligible (Figure S2). The autocatalytic production of IM2 (40–60 min) is accompanied by a significant fall in the CD_305_ signal to negative values. The ellipticity CD_305_ returns to zero after 12 h from the beginning of the reaction (not shown), and no significant CD signal in the region of 260–450 nm can be detected. This observation indicates the absence of intermediates IM1 and IM2 at the end of the reaction.

The absorption spectrum of the reaction solutions after the completion of the reaction (12 h) shows a maximum at 310.6 nm (yellow line in Figure 8b, end-spectrum), in agreement with the maximum of protonated ABTS, HABTS^−^ (310 nm [55], see also Figure S3). Although the HABTS^−^ absorption overlaps the 302 nm band of IM1, a quantitative assessment of the IM1 consumption upon the ABTSr addition can be made, as illustrated by Figure 8b. Immediately before the ABTSr addition, the 302 nm band is developed (magenta line; see also the inset), reaching the *A*_300_ absorbance of 0.178. Immediately after the ABTSr addition (16 min), the absorption in the 260–380 nm region significantly increases due to the formation of HABTS^−^. The composed spectrum (blue line) represents the sum of the HABTS^–^ spectrum and the spectrum of the residual IM1 that has not been consumed by the reaction with ABTSr. The absence of absorption peaks above 400 nm (see also the 16–14 min difference spectrum, red line) proves that no residual ABTSr is present, and its total conversion to HABTS^−^ can be assumed. The contribution of HABTS^−^ can be simply eliminated from the composed spectrum at 16 min by subtraction of the end-spectrum. The resulting spectrum (the light blue line in the inset) corresponds to the spectrum of the residual IM1 after the ABTSr addition with *A*_300_ = 0.044. The negative difference spectrum obtained as 16 min–end–14 min spectrum (green line) then documents the consumption of a species showing the 302 nm band. A comparison of inversion of such a difference spectrum with the spectrum at 14 min (green line vs. magenta line in the inset) confirms the identification of the consumed species with IM1. Thus, we conclude that ABTSr, when initially present or added to the reaction solution, immediately accepts an electron from the first intermediate and turns to its reduced and protonated form. The comparison of *A*_300_ values gives an estimate for the degree of conversion of IM1 as 75%, in agreement with the less accurate estimate from the CD record (Figure 8a). Providing that the IM1 consumption is due to the stoichiometric conversion of 20 μM ABTSr to HABTS^−^, the molar attenuation coefficient of IM1 per copper ion can be estimated as *ε*_300_(IM1/Cu) ≈ 7.7 × 10^3^ M^−1^ cm^−1^.

**Figure 9:**
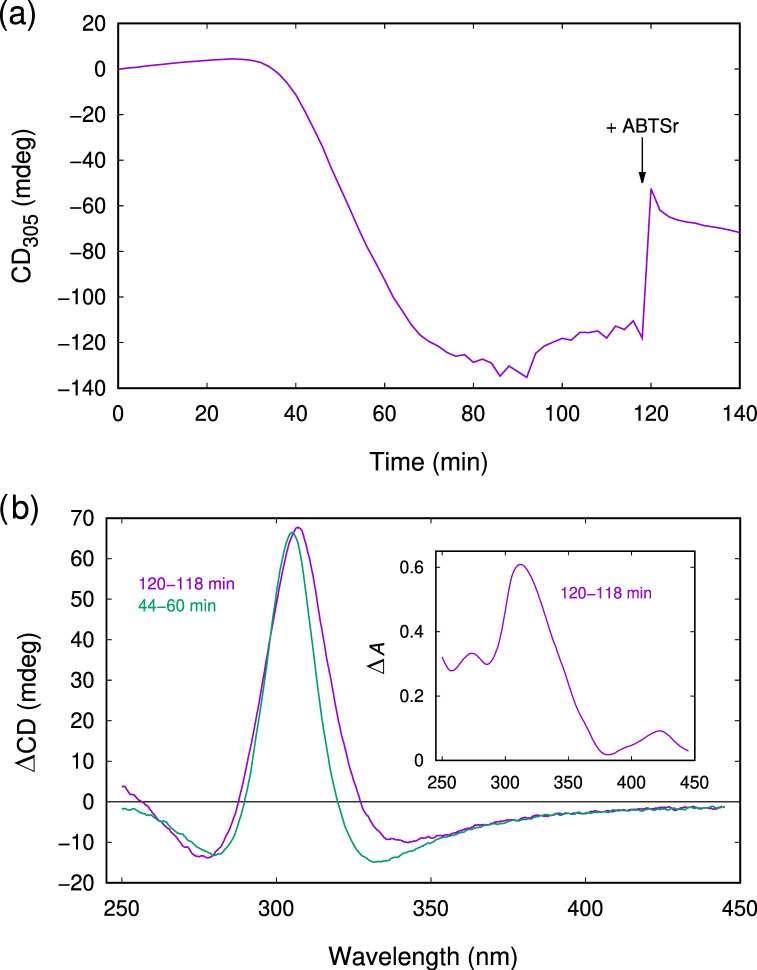
CD and UV/Vis spectrophotometric analysis of the effect of ABTSr addition (at 118 min) during the reaction of 300 μM Cu^2+^ with 300 μM NAC in 40 mM HClO_4_ at 20 °C. The spectra (445 → 250 nm, resolution 0.2 nm) are scanned in 2 min cycles at a rate of 100 nm min^−1^. (**a**) A time record of the ellipticity at 305 nm; 20 μM of ABTSr is added at 118 min (arrow) from the beginning of the reaction. The ABTSr addition causes a decrease (in absolute value) in the CD_305_ signal of about 55%. (**b**) Difference CD and UV/Vis (inset) absorption spectra plotted as difference ellipticity (ΔCD) and difference absorbance (Δ*A*) vs. wavelength. Details are explained in the text.

The second intermediate also proves to be an effective scavenger of the ABTSr. If ~20 µM of ABTSr is added to the reaction solution with the significant IM2 accumulation (>100 min), the free radical immediately converts to its reduced form (Figure 9). The negative ellipticity at 305 nm is decreased to 55% in its absolute value upon the ABTSr addition (Figure 9a). This change corresponds to the consumption of a species with the Cotton effects characteristic for the second intermediate, as demonstrated by a difference CD spectrum (Figure 9b, the magenta line) obtained by subtraction of the spectrum scanned at 118 min (immediately before the ABTSr addition) from the spectrum scanned at 120 min (immediately after the ABTSr addition). This difference spectrum compares well with a difference CD spectrum (the green line) obtained by subtraction of the spectrum scanned at 60 min from the spectrum scanned at 44 min. As the period of 44–60 min is characterized by the autocatalytic production of IM2, the similarity of the difference CD spectra points to IM2 as the substance involved in the reaction with ABTSr. The small variance in the spectra (the 305 nm peak of the consumed species is broader and red-shifted) can be explained by further modification (e.g., polymerization) of IM2 during the reaction. The difference (120–118 min) absorption spectrum (the inset in Figure 9b) indicates the conversion of ABTSr to HABTS^−^ upon its addition (compare with the red line in Figure 8b); however, the small absorption in the region of 380–450 nm indicates the possible presence of residual ABTSr. This absorption is not detected in the next spectral scan after 2 min.

## 3. Discussion

The presented results report that the interaction of cupric ions with NAC in acidic aqueous solution leads to the formation of relatively stable intermediates, which can be conveniently followed by UV/Vis absorption and CD spectroscopies. These intermediates are capable of fast electron transfer to the free radical derived from ABTS and most probably are the main catalytic species significantly facilitating the oxidation of NAC by ABTSr (Figure 1) and, accordingly, other cysteine-derived thiols, as previously reported [52].

However, these findings prompt numerous questions regarding the structure, properties, and reactivity towards dioxygen and ROS with respect to the possible biological activity of such intermediates. Although additional research is needed to address these important questions in detail, the obtained data allow for preliminary considerations within the context of the previous knowledge that has been collected over the past several decades.

### 3.1. Interpretation of the Absorption around 300 nm

First, we examine the spectral data reported above in relation to the hypothetical structures of the proposed intermediates. An assignment of the observed absorption band centered at 302 nm ascribed to the first intermediate IM1 could be instructive for structure predictions. Unfortunately, this spectral region comprises transitions of various types, some of them are still discussed. Possible candidates for IM1 include free radicals derived from NAC or its disulfide, diNAC. The cysteine thiyl radicals (RS^•^) show an absorption maximum at 340 nm, but a low molar attenuation (extinction) coefficient, *ε*_340_ = 140 M^−1^ cm^−1^ [56], excludes this type of species. Moreover, the RS^•^ radicals are expected to accept electrons rather than to reduce ABTSr. The cystine radical anions (RS^•^–SR^−^) absorb intensively (*ε*_420_ = 7600 M^−1^ cm^−1^ [56]), but the absorption maximum, 420 nm, is too shifted from 302 nm to match with IM1.

The elimination of uncoordinated sulfur radicals takes copper complexes into account as a more feasible suggestion for the observed intermediates. Although complexes of thiols, particularly those derived from cysteine, with both Cu^+^ and Cu^2+^ ions have been studied for a long time, uncertainties still exist about their exact composition, stability, and properties, including the spectral characteristics. Cu(I)–thiolates–Cu(RS), [Cu(RS)_2_]^−^, or polynuclear clusters and polymers, are supposed to be stable due to strong Cu–S bonding [26,32,57], but colorless, showing no significant absorption above 300 nm. On the contrary, Cu(II)–thiolates are spectroscopically active in the range of 300–350 nm, but usually unstable, subject to electron transfer from sulfur to copper. Several transients have been reported for reactions of Cu^2+^ with various thiols with the following absorption maxima or shoulders (in nm): 300, 330, and 340 for cysteine as the substrate [29,31,35,36,39,58,59]; 333 and 340 for penicillamine [59,60]; 300, 340, and 350 for 2-mercaptosuccinic (thiomalic) acid [34,61]; 303, 330, and 350 for NAC [62,63]; 300 and 297 for GSH [37,43,64]. Although electronic structures of such intermediates are not clear, many authors accept that copper is in the oxidation state +2 and the observed absorption bands arise from ligand to metal charge transfer (LMCT) transitions from the coordinated thiolate anion to Cu(II). However, precise assignments of the transitions are difficult, as distortion from the square planar geometry of the complexes can significantly shift the absorption maximum toward longer wavelengths [65,66]. In a few cases, 300 nm shoulders were attributed to Cu(I)–thiolates [36,37,64]. Mixed-valence Cu(I)–Cu(II) complexes with bridging thiolates absorbing at 520 nm [27,33] and 340 nm [59] have also been proposed. Such a complex with penicillamine is a rare example when its structure was successfully determined [33]. A superoxide-bridged Cu(I,II) mixed-valence intermediate in the reaction of cysteamine or cysteine with Cu^2+^ has been suggested by Sakurai et al. [67]. Additional hypothetical transients possibly formed during copper–thiol interactions include complexes of Cu(I) or Cu(II) with thiyl radicals [18,30,35,36,43,68,69,70].

The formation of Cu(II)–thiolates is very fast, as confirmed by rapid mixing and stopped-flow techniques [29,31,62,71,72], and usually, only decay of such initially formed intermediates can be followed. This is consistent with the associative substitution mechanism for ligand exchange in metal complexes [73]. The rate constant of 5.7 × 10^9^ s^−1^ for the water exchange reaction of the Cu^2+^ aqua ion has been reported [74]. Thus, the water molecule exchange for a thiol ligand is expected to proceed with a second-order rate constant not significantly lower than 10^8^ M^−1^ s^−1^. Consequently, it can be predicted that a Cu(II)–NAC complex should be formed during an initial period shorter than a millisecond under the conditions of our experiments. Such an expectation is, however, in stark contrast to the observed production rate of IM1 (Figure 2 and Figure 4). Additionally, the experiments with additions of ABTSr to the reaction solutions show that both IM1 and IM2 act as electron-donors, suggesting that these intermediates are Cu(I) or mixed-valence complexes. Taking into account that Cu(I)–thiolates absorb light below 300 nm and Cu(I)–Cu(II)–thiolates above 500 nm, it is unlikely that the intermediates observed in our system could be identified with complexes of such types.

We incline to a hypothesis that the intermediates detected in the studied reaction—and possibly also in other systems containing thiols and copper ions—are dicopper–disulfide complexes of the type Cu(I)–S–S–Cu(I). Although only limited information exists on the stability and properties of such complexes, several examples have been prepared and characterized [38,75,76,77,78,79]. Importantly, such complexes show UV/Vis spectra with shoulders around 300 nm, which are interpreted as a metal to ligand charge transfer (MLCT) from Cu(I) to disulfide, or as a disulfide absorption significantly red-shifted due to Cu complexation and/or a deviation of the CSSC dihedral angle from 90° [75]. The values for the molar attenuation coefficient of such transitions of the order of 10^3^ M^−1^ cm^−1^ agree with our observations. Charge transfer (CT) transitions are usually intense due to relatively high values of the transition dipole moment, and if several such chromophores are appropriately positioned in the molecule, a band split arising from the interaction of the transition moments (exciton coupling) is observed. For example, a transition dipole-vector coupling has been described in Cu(II)–O–O–Cu(II) complexes [80] including oxy-hemocyanin [81]. If the interaction energy of the transition dipoles is low, the band split is not recognized in an absorption spectrum, but is readily seen in a CD spectrum, as the original transition splits into two Cotton effects of opposite signs with equal absolute values of the rotational strength [54].

### 3.2. Interpretation of the CD Spectra and Proposed Structures of the Intermediates

**Chart 1:**
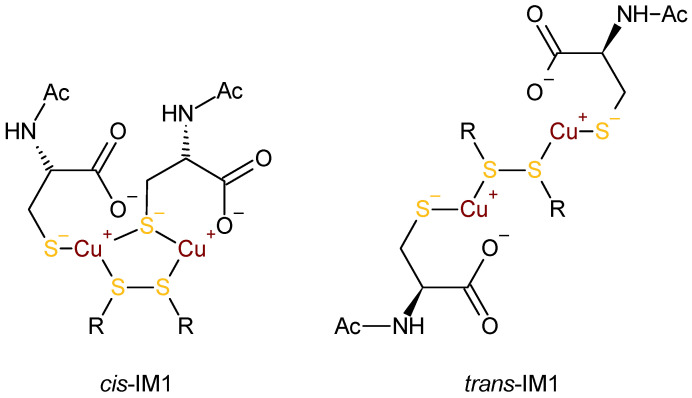
Proposed structures for stereoisomers of the first intermediate, IM1. Ac denotes the acetyl group and R is the NAC moiety without the thiol group.

The CD spectrum in the left panel of Figure 5, assumed to be close to the first intermediate, shows a split into the two maxima at 284 and 302 nm, but only positive Cotton effects are observed. This fact seems to be inconsistent with the above hypothesis that the 302 nm band arises from the Cu(I) → disulfide MLCT, as both positive and negative signals would then be expected due to exciton coupling of the two chromophores. However, this discrepancy provides further evidence that the first intermediate consists of two or more similar compounds, as suggested above based on deformations seen in the difference spectra (Figure 6, left). We propose that IM1 is present in two forms distinguished as *cis*- (c-IM1) and *trans*- (t-IM1) stereoisomers (Chart 1), which are initially formed in similar amounts.

We assume that the observed CD spectrum (Figure 5, left) originates from the S–S bond axial chirality of both isomers [82]. Since the existence of axial chirality requires the restriction of rotation about the axis of chirality, it can be deduced that the rotation about the S–S bond is hindered in the IM1 complex, probably due to steric effects and possible stabilization of c-IM1 by one or two thiolate bridges. Thus, each of the isomers possesses a different Cu–S–S–Cu dihedral angle, which determines the signs of the Cotton effects resulting from the respective exciton split in each isomer [54]. The observed CD spectrum is then a net sum of mutual compensation of the positive and negative Cotton effects in both isomers (Figure S4). Note that the sum would not be exactly zero even for the equal concentrations of the isomers, as they are not true optical antipodes (in terms of M-, P-types), but disulfide rotamers (atropisomers) with the Cu–S–S–Cu dihedral angles that may not be equal in their absolute values. The optical antipodes of both IM1s would be the result of coordination with d-NAC and d-diNAC, as the axial chirality of IM1s is given by the asymmetry of the ligands.

**Chart 2:**
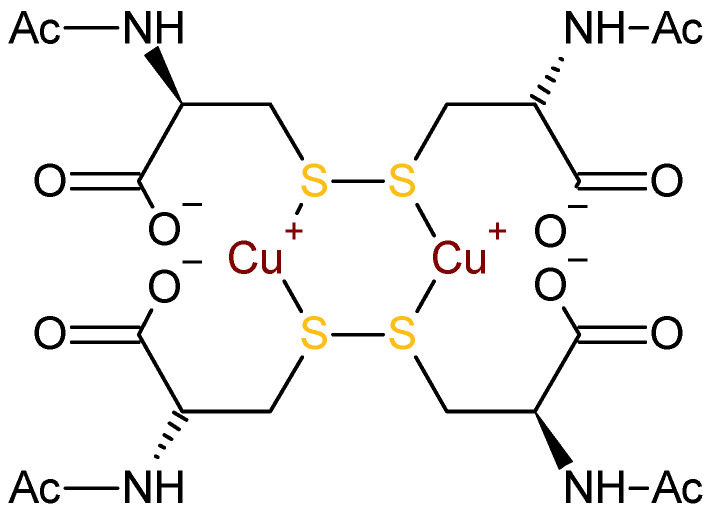
Proposed structure for the second intermediate, IM2. Ac denotes the acetyl group.

The production of IM2 is accompanied by the consumption of one of the IM1 isomers, presumably c-IM1, while the concentration of another one (t-IM1) still increases, as deduced from the difference absorption spectra (Figure 6, left). This observation can be explained by assuming that only the conformation of c-IM1 is suitable for further conversion to IM2. Accordingly, the structure of IM2 is proposed as another dicopper complex coordinated with two disulfide ligands (Chart 2).

The proposed structure of IM2 allows several combinations for coupling of the transition dipole moments due to the Cu(I) → disulfide MLCT transitions. Such a multiple coupling could account for a more complex exciton split of the original Cotton effect, as seen in the CD spectrum of IM2 (Figure 6, right). As mentioned above and corroborated in the Supplementary Materials (Figure S5), the three Cotton effects satisfy the sum rule (the sum of their rotational strengths is zero), suggesting that the strong negative Cotton effect centered at 305 nm consists of two similar or identical exciton splits. The presence of another disulfide in the IM2 molecule is consistent with the delayed production of IM2 in comparison with IM1.

A lower limit estimate for the molar attenuation coefficient of IM1 can be calculated based on the absorption at the time of maximum in the IM1 concentration (Figure 4). Providing that all the present copper is bound to the IM1 complex and the contribution of other species to the absorption at 302 nm is negligible, a value of ε_302_ > 3.5 × 10^3^ M^−1^ cm^−1^ is obtained. A more accurate estimate is gained from the change in the 302 nm absorbance upon stoichiometric conversion of the added ABTSr to HABTS^–^ (Figure S2). Assuming the proposed structure for IM1 (Chart 1) and two single-electron transfers from IM1 to two molecules of ABTSr, an evaluation yields ε_302_ > 8 × 10^3^ M^−1^ cm^−1^ per Cu ion. This value also represents a lower limit, as other species could react with ABTSr simultaneously with IM1, or IM1 could be consumed due to depletion by ABTSr of a species that is in a fast equilibrium with IM1.

The obtained CD spectra allow estimating the anisotropic dissymmetry factor *g* [83] that provides further insight into the structures of the detected intermediates. The *g*-factor equals to (*ε*_L_–*ε*_R_)/*ε*, where *ε*_L_ and *ε*_R_ are the molar attenuation coefficients for the left and right circularly polarized light, respectively. Absolute values of the dissymmetry factors are usually of 10^−3^ order of magnitude for small organic molecules in isotropic bulk solutions [84]. A value of *g*_302_ ≈ 10^−3^ is found for the first intermediate. Considering the assumption that the observed CD signal is substantially reduced by mutual compensation of the contributions from the two isomers, it can be expected that absolute *g*-factors of the individual isomers are significantly higher than 10^−3^. This fact indicates a higher anisotropic dissymmetry than is usually observed for intense absorption bands, which we attribute to the restriction of rotation around the disulfide bond. Although the molar attenuation coefficient of IM2 is not evaluated from our data, it is clear that the value for the region around 300 nm is lower than ε_302_ for IM1, as the production of IM2 is accompanied by the decrease in the absorbance (Figure 7a, magenta line). At the same time, the absolute value of the CD signal increases (Figure 7b), so it can be deduced that a |*g*_305_| value for IM2 considerably exceeds 10^−3^, as well. An estimate based on the difference spectra (Figure 6) suggests a value even higher than 10^−2^. This is consistent with the proposed structure of IM2 with a fixed Cu–S–S–Cu dihedral angle. In addition, a polymeric form of IM2 is expected to show an enhanced anisotropy in comparison with the monomers in a bulk solution.

### 3.3. The Proposed Intermediates as Oxidation Catalysts

Important information on the mechanisms governing the behavior of the complex system under study provides the sigmoidal production kinetics of the second intermediate (Figure 7). As suggested above, the observed time courses indicate autocatalysis, implying IM2 as an effective catalyst. Based on the proposed structure of IM2, it can be assumed that its production depends on a sufficient amount of disulfide produced. Consequently, IM2 is likely to be a strong candidate for the main catalyst in the autoxidation process. Kachur et al. [18] observed sigmoidal kinetics of dioxygen consumption in a second reaction phase of copper-catalyzed autoxidation of NAC at pH 7.4. Although the authors considered the Cu^II^(RS)_2_ complex proposed by Cavallini et al. [29] as the reaction catalyst in the autoxidation of cysteine and its derivatives, they pointed to a possible role of disulfide in the binding of copper ions. Interestingly, a catalytic effect of the oxidized glutathione (disulfide) on the sigmoidal consumption kinetics of methylene blue in a reaction with glutathione at pH 7 (also with cysteine or thioglycolic acid) was reported almost a century ago [85]. Sigmoidal kinetics interpreted as autocatalysis by Cu^2+^ ions have also been observed by Buzuk et al. [42] in the autoxidation of cysteine in the presence of cupric ions (pH 5–8). Similarly, rapid decay of the intermediate showing absorption maximum at 330 nm in a final phase of the alkaline cysteine autoxidation [29] seems to follow a sigmoidal course. Interestingly, this phase of the reaction is accompanied by a sudden disappearance of accumulated hydrogen peroxide, ascribed by authors to the catalytic action of liberated Cu^2+^ ions [86]. Sigmoidal decay kinetics of species absorbing at 260 and 300 nm have been reported by Pecci et al. [36], who studied copper-catalyzed oxidation of cysteine at pH 7.4.

Additional considerations based on the proposed structures of IM1 and IM2 further support the hypothetical role of these intermediates as oxidation catalysts. Binuclear Cu(I) complexes are comprehensively studied for their ability of binding dioxygen to form Cu(II) peroxo-complexes [87] in a biomimetic approach to understanding the oxidase activity of copper-containing enzymes [88,89,90,91]. In particular, a few examples of dicopper–disulfide complexes as possible catalysts involved in oxidation reactions exist in the literature. Ohta et al. [77] described the synthesis of a yellow disulfide-bridged dicopper(I) complex with an absorption maximum at 300 nm attributed to Cu(I) → disulfide MLCT. The complex catalyzed the oxidation of cyclohexane in the presence of H_2_O_2_, probably via a Cu(II)–OOH intermediate. Lee et al. [92] showed that oxygenation of a Cu^I^_2_–disulfide complex leads to oxidation of the disulfide ligand to a sulfonate. An exceptionally high catecholase activity of a binuclear Cu(II)–disulfide complex has been reported by Ording-Wenker and coworkers [41]. The authors hypothesized a catalytic role of a Cu^I^_2_–disulfide intermediate reacting with dioxygen to form a side-on Cu^II^_2_(*μ*-*η*^2^:*η*^2^-peroxide) species analogous to oxy-hemocyanin.

**Chart 3:**
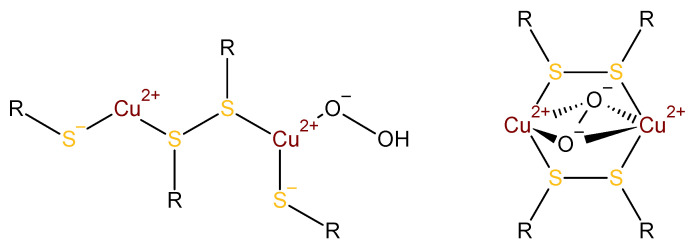
Proposed dioxygen adducts of t-IM1 (**left**) and IM2 (**Right**).

The possibility that the proposed intermediates IM1 and IM2 could bind dioxygen to form peroxo-complexes as illustrated in Chart 3 is intriguing and seems to be feasible. Various adducts of copper species with the dioxygen molecule have been proposed as hypothetical intermediates in the autoxidation of thiols [35,67,68,72]. Yamauchi and Seki [93] suggested transient adducts of dioxygen with binuclear Cu(I) or mixed-valence Cu(I)–Cu(II) complexes of disulfides in a mechanism of Cu(II)-catalyzed disulfide bond cleavage to sulfinate. The main difficulty that needs to be overcome to capture dioxygen to a binuclear Cu complex is the significant negative change in entropy associated with such a process [94]. In this respect, bridging two Cu(I) ions by a disulfide would be profitable. The SS bond is flexible due to its high polarizability and the ligation of sulfur to Cu(I) stabilizes the structure. In particular, the coordination of the second disulfide in the IM2 suppresses the rotation of Cu atoms around the SS bond and fixes them in a position suitable for trapping the dioxygen molecule. As the Cu–Cu distance (3.9 Å) in dicopper–disulfide complexes [38,79,95] is close to that (3.6 Å) in oxy-hemocyanin [96], the same side-on mode for dioxygen binding is expected. Coordination of the four carboxylate anions of the two diNACs with Cu^2+^ ions effectively compensates for the positive charge generated at the catalytic center upon the dioxygen molecule binding. Interestingly, it has been shown for the multicopper oxidases that redox properties of the trinuclear Cu cluster that reduces O_2_ to H_2_O are tuned by four anionic carboxylate residues within ~10 Å of the cluster [97]. Another advantage of a disulfide bridge for binuclear catalysts may be electron transfer between the copper ions. Thus, for example, IM1 could reduce O_2_ directly to H_2_O_2_ in two successive one-electron steps, preventing a release of superoxide. Notably, a polymeric form of a dicopper–disulfide complex has been described [76]. Such a polymeric electronically coupled chain could function as an extremely efficient oxidation catalyst. Sharma et al. [63] reported the formation of nanoparticles during oxidation of NAC by methylene blue catalyzed by Cu(II) in hydrochloric acid and anticipated a gel-like Cu–NAC network [98] acting as the catalyst.

### 3.4. Mechanistic Considerations

The concept of a possible formation of binuclear disulfide–dicopper(I) complexes in reactions of thiols with Cu^2+^ ions had been proposed as early as 1966 by Hemmerich et al. [28]. It was originally assumed that the initially formed bis(*μ*-thiolato)dicopper(II) complex is chemically indistinguishable from the isoelectronic Cu^I^_2_–disulfide:

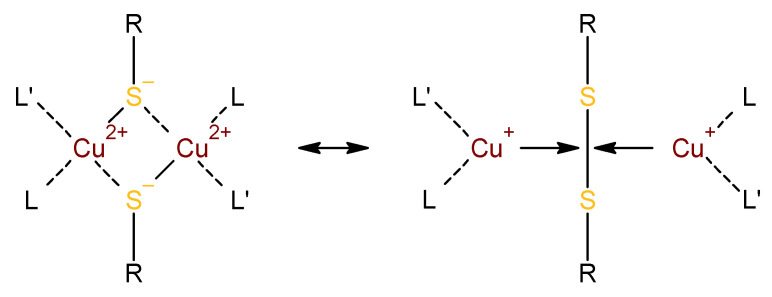



The present knowledge proves the existence of both complexes, which can interconvert [38,78,79,99,100]. The equilibrium is shifted to the left or right depending on the thiol structure and other factors such as temperature, pH, and the presence of chloride ions. Available kinetic data [71] suggest a dimerization step at the initial phase of reactions of Cu^2+^ with various thiols, including cysteine in aqueous ammonia (pH 11.5). The observed second-order decay of presumably a Cu^II^(RS)_2_ complex has been interpreted as the production of a bis(*μ*-thiolato)dicopper(II) transient followed by a concerted reduction of copper(II) and disulfide formation [37,71]. Lappin et al., in a kinetic study [34], proposed acid-catalyzed dimerization of a transient Cu^II^RS complex with an absorption maximum at 350 nm in a reaction of 2-mercaptosuccinic acid with Cu^2+^ in aqueous perchloric acid (pH 1.4–4). The dimer Cu^II^_2_(RSH)(RS) undergoes redox conversion upon attack of another thiol molecule producing disulfide and a yellow binuclear Cu^I^–thiolate complex. The exact nature of the yellow product was not investigated, but the authors assumed that it was likely a dimer that was subsequently oligomerized. We note that the reported substantial absorption above 300 nm of the product allows for the possibility that at least partial coordination of Cu(I) by disulfide is involved. The work of Lappin et al. [34] pointed out for the first time that a binuclear Cu intermediate could provide a template on which direct two-electron oxidation of thiols to disulfides can occur, circumventing the formation of high-energy radicals. A disulfide-bridged dicopper(I) complex stabilized by an auxiliary ligand 2,2′-bipyridine has been suggested as an intermediate in the autoxidation of d-penicillamine by Seko et al. [40]. Interestingly, a different mechanism leading to the known Cu(I)–Cu(II) mixed-valence 14-nuclear cluster [33] applies when ethylenediamine is used as the auxiliary ligand [40].

The kinetics of ABTSr reduction by NAC in the presence of Cu^2+^ (Figure 1) conform to the proposed formation of binuclear copper complexes. Although a detailed kinetic and mechanistic analysis of the reaction is in progress, preliminary results have shown that the observed time courses can be interpreted as a coincidence of zero- and first-order decay of ABTSr. The zero-order pathway is governed by second-order kinetics to Cu^2+^ ions. This is consistent with previous findings on Cu-catalyzed autoxidation of thiols. Lee and Notari [101] observed a transition from first to zero order for captopril oxidation rates upon a decrease in the thiol concentration at pH 6.6−8. Importantly, a second-order dependence of the zero-order rate constant on cupric ion concentration was found. Similarly, Ehrenberg et al. [47] reported second-order kinetics with respect to Cu^2+^ for oxidation of cysteine by dioxygen at pH 8.1 and suggested the involvement of binuclear complexes, or collisions between two mononuclear complexes, in the catalytic action of copper. Bagiyan et al. [70,102] demonstrated a zero-order dioxygen consumption rate with a second-order dependence on Cu^2+^ or Cu^+^ for cysteine and cysteamine autoxidation in alkaline aqueous solutions. A second-order term with respect to Cu^2+^ in the dioxygen consumption rate for alkaline cysteine autoxidation was earlier detected by Zwart et al. [35], who proposed a dioxygen-bridged dimer of Cu^II^(RS)_2_ as the key intermediate.

**Scheme 1:**
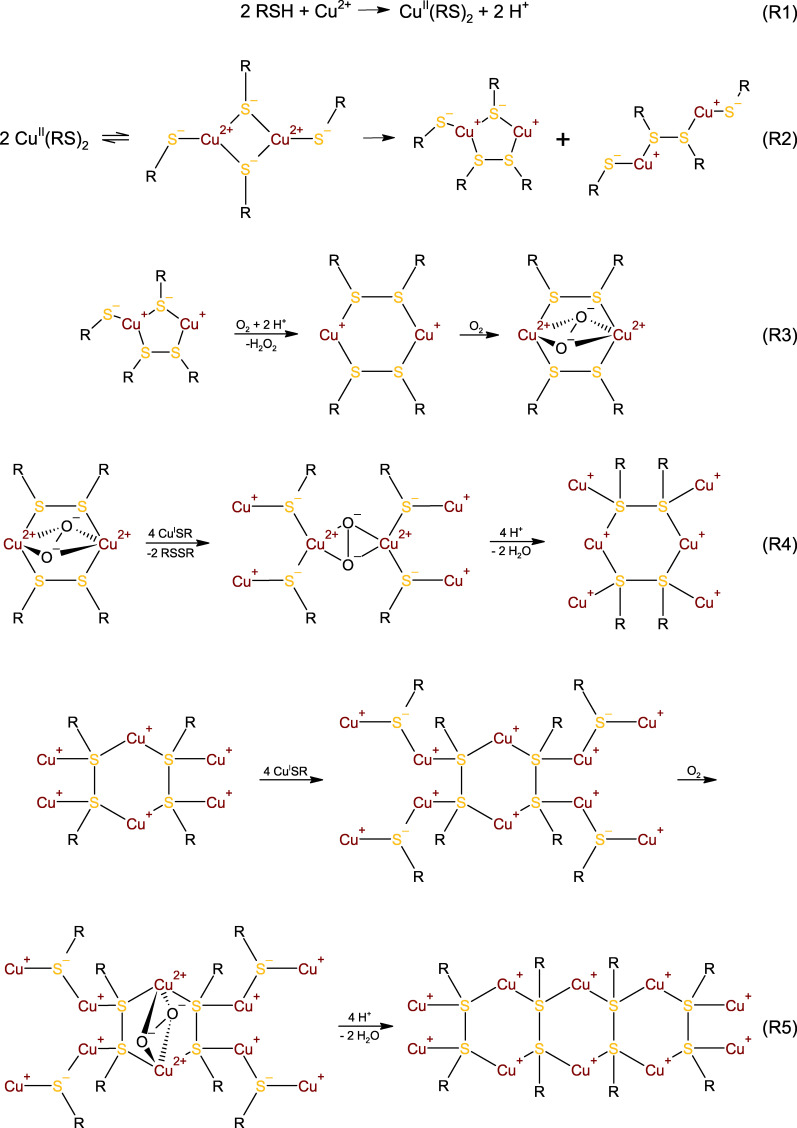
Proposed mechanism of the formation and catalytic activity of the second intermediate.

Further research is needed to explore the detailed mechanism of the studied reaction. The observations described above are in agreement with the former assumption that an initial step of a thiol interaction with Cu^2+^ is the formation of the Cu^II^(RS)_2_ complex (Scheme 1, R1). The fate of the complex depends on many factors, e.g., its structure and redox potential, initial concentrations and ratio of the reactants, pH, presence of O_2_ and Cl^–^ (or other ligands), and obviously, simultaneous reaction pathways can occur. An instant electron transfer from sulfur to copper leads to the formation of Cu(I)–thiolates, which react with ABTSr according to pseudo-first-order kinetics, as previously suggested [52]. This pathway is dominant at high ratios of the thiol over the cupric ions. A parallel pathway showing zero-order kinetics with respect to ABTSr becomes significant at lower RSH/Cu^2+^ ratios. The second-order kinetics with respect to Cu^2+^ are consistent with dimerization of the Cu^II^(RS)_2_ complex followed by a slow interconversion to form both the isomers of IM1 (Scheme 1, R2). Subsequent rapid scavenging of ABTSr by IM1 produces ABTS, Cu^2+^, and disulfide. Providing that the interconversion is the rate-limiting step, the consumption rate of ABTSr is independent of its concentration for this pathway, in agreement with the observations (Figure 1). The deviation of the reaction rate from a straight line indicates a simultaneous action of both pathways.

Oxidation of the proposed intermediates by atmospheric dioxygen is assumed to occur in the absence of ABTSr. A one-electron transfer from Cu(I)–thiolates to dioxygen producing superoxide O_2_^•−^ (or HO_2_^•^ in acidic medium) is expected [36]. The intermediate IM1 is supposed, as considered above, to convert dioxygen directly to H_2_O_2_ in a second reaction pathway. Notably, two simultaneous processes (superoxide-dependent and peroxide-dependent) have been suggested by Kachur et al. [18] to explain two-phase kinetics of dioxygen consumption and hydroxyl radical production in a copper-catalyzed autoxidation of cysteine and its derivatives. Both processes are involved in the first phase with a constant dioxygen uptake. The second phase begins after a substantial consumption of the free thiol (when unoxidized thiol is twofold higher than copper in the case of NAC and equimolar in the case of cysteine) and is characterized by an intense production of ^•^OH radicals in the peroxide-dependent process. Interestingly, the second phase shows sigmoidal kinetics when NAC [18] or GSH [103] is used as the substrate. Although it is accepted that Cu(I) species can react with H_2_O_2_ to produce ^•^OH radicals in a Fenton-like process [17], the role of ^•^OH radicals in thiol autoxidation is not clear. Kachur et al. [103] used hydroxylation of coumarin-3-carboxylic acid for ^•^OH detection; however, an alternative Cu-catalyzed hydroxylation of the probe should be considered. As mentioned above, hydroxylation of cyclohexane and cyclohexene by means of a disulfide-bridged dicopper(I) complex in the presence of H_2_O_2_ has been reported [77]. Cu-catalyzed hydroxylation of the aromatic ring might also be possible, as exampled by the enzyme tyrosinase possessing a coupled binuclear (type 3) Cu active site [96].

The sigmoidal phase of the studied reaction characterized by an increase in the intensity of the negative CD_305_ signal (Figure 7b) can be ascribed, as suggested above, to an autocatalytic production of the intermediate IM2. We assume that it is initiated by a relatively slow oxidation of c-IM1, which assembles an effective catalyst IM2 capable of activating dioxygen by forming a side-on peroxide-bridged dicopper(II) complex (Scheme 1, R3). As the affinity of Cu(II) to disulfides is probably significantly lower than to thiolates, the disulfides are readily substituted by IM1 or Cu^I^RS in the peroxo-complex. Following electron transfers from the thiolate, sulfur atoms reduce peroxide to water and Cu(II) to Cu(I). The transiently formed thiyl radicals immediately couple at the binuclear template, and the ring Cu^I^_2_(RSSR)_2_ catalytic unit is reassembled (Scheme 1, R4). The four Cu(I) ions at both ends of the cluster form appropriate binding sites for other electron-donating substrates, while the two Cu(I) ions in the ring are set up for trapping another dioxygen molecule. The electrons from the substrates (Cu^I^RS) are transferred to the catalytic center through the S–Cu–S–Cu pathway, and the four-electron reduction of dioxygen to water results in the propagation of the cluster (Scheme 1, R5). Such a mechanism resembles the electron transfer from a substrate-binding Cu center (type 1) to a catalytic trinuclear Cu cluster (type 3) via a superexchange cysteine-histidine pathway [97] in multicopper oxidases. The propagation rate would increase with the length of the –[Cu^I^_2_(RSSR)]*_n_*– polymer due to the increasing number of catalytic centers. Thus, the autocatalytic progression of the CD signal attributed to the Cu^I^_2_(RSSR)_2_ chromophore in the monomers or various oligomers of IM2 can be explained. The electron transfer from the binding site to the catalytic center could occur through a relatively long distance in the growing polymer, as a possible shift in *π* density from Cu(I) to S atom has been discussed for Cu(I)–disulfides [75]. A similar mechanism could also apply to the oxidation of polymeric thiolates [Cu^I^SR]*_x_*. Autocatalytic oxidation of Cu^I^SR in a final phase of cysteine autoxidation at pH 5 has been proposed by Buzuk et al. [42].

In addition to O_2_, the proposed polynuclear Cu(I)–disulfide clusters are likely to utilize H_2_O_2_ as an oxidant for two-electron oxidations. Such an oxidation reaction could convert H_2_O_2_ to water without initiation of the Fenton process, as suggested for Cu(I)–glutathione aggregates [37].

The sigmoidal phase ends when the thiolate sulfur is converted to disulfides, and slow degradation of the Cu(I)–disulfide complexes follows. This process is probably in part due to Cu(I) autoxidation producing Cu^2+^ and the disulfide (possibly associated in a Cu(II)–RSSR complex) and in part due to ligand decomposition by self-oxidation, resulting in the sulfoxidation of disulfides to sulfinates and sulfonates, as previously described for complexes of this type [77,92,93]. The formation of sulfonates in the second phase of the copper-catalyzed autoxidation of cysteine and its derivatives has been proposed [18]. The degradation of the Cu(I)–disulfide complexes is O_2_-dependent (Figure 4b) and could be aided by a UV photodissociation of the disulfide bonds [104], in agreement with the observed effect of light on the decay rate of IM1 (Figure S1). Importantly, a cleavage of one of the disulfide bridges in a Cu^I^_2_(RSSR)_2_ complex upon photoexcitation-induced CT has been recently described [95].

### 3.5. An Alternative: Cu(I)–Thiolates as Catalytic Intermediates

An alternative explanation of the nature and properties of the observed transients provides the possibility that IM1 and IM2 identify with Cu(I)–thiolates rather than with Cu(I) disulfides. As Cu(I)–thiolates can also form binuclear or polynuclear complexes [105,106,107,108,109], this alternative hypothesis could explain the proposed catalytic activity of IM1 and IM2 in a similar way as the disulfide hypothesis. As mentioned above, such intermediates have been proposed as possible oxidation catalysts [34,37]. Moreover, the observed UV/Vis and CD spectral features may be due to the CT transition between the Cu and thiolate S atoms or other CT (e.g., from Cu to carboxylate ligand or to the solvent) with the possible coupling of the transition dipole moments in clusters of a higher nuclearity. Although both hypotheses should be examined in further research, we present several arguments in favor of the disulfide hypothesis.

First, the assignment of the observed spectral bands around 300 nm to Cu(I) → S–S MLCT transition is supported by examples of the synthesized compounds [75,76,77], while similar CTs in Cu(I)–thiolates have less evidence. Although both LMCT [110] and MLCT [107] transitions have been suggested for Cu(I)–thiolate clusters, such assignments remain uncertain. For illustration, Vortisch et al. [32] studied the properties of a series of cuprous thiolate complexes with 16 ligands (e.g., cysteine and GSH) in various forms including polymers, and concluded that electronic spectra of these complexes do not show any significant absorption maxima both in the UV and visible regions. On the contrary, Pecci et al. [36] attributed a peak at 260 nm and a shoulder at 300 nm produced upon the addition of 200 μM Cu^2+^ to a solution of 2 mM cysteine at pH 7.4 to a Cu^I^(RS)_2_ complex. Similarly, an absorption shoulder at 300 nm observed in Cu^2+^/GSH systems was attributed to a Cu(I)GS complex [37] and to a tetranuclear Cu^I^_4_(GS)_6_ cluster [64]. Such attributions in the Cu^2+^/RSH systems have been supported by evidence that corresponding Cu^+^/RSH systems show similar or even identical electronic spectra in the wavelength region under consideration. However, ensuring the absence of disulfides in Cu^+^/RSH systems is difficult in practice. Even though the spectrophotometric measurements of Cu(I) complexes are usually performed in strictly anaerobic conditions, traces of residual dioxygen in the samples should be considered at a lower limit of 0.2–0.4 ppm [111]. Such a concentration of O_2_, if reduced to H_2_O_2_, may produce disulfide at a concentration of more than 10 μM. Assuming a complete ligation of the produced disulfide with Cu^I^RS to form a complex as proposed above (Chart 1), a contribution to the absorbance *A*_300_ > 0.15 can be expected. The concentration of disulfides in commonly studied Cu^+^/RSH systems is probably even higher due to a possible four-electron reduction of residual O_2_ to H_2_O and due to the disulfide impurities usually present in the starting material [112], particularly if the thiol is used in a high excess over the Cu(I) concentration. Accordingly, an alternative that the spectral activity around 300 nm observed in such systems arises rather from Cu(I) interaction with the disulfide than exclusively from Cu(I)–thiolate complexes should be examined.

Interesting data on the Cu(I)–thiolate interactions are provided by studies of Cu metallothioneins (Cu-MTs)–metalloproteins, which are assumed to coordinate Cu(I) with cysteine residues forming various polynuclear Cu clusters. Similar to low-molecular-weight thiols, prominent shoulders at around 262 and 295 nm were observed as metal-dependent features in the absorption spectra [113,114]. The high-energy band (262 nm) was assigned to the CysS → Cu(I) LMCT transition, possibly mixed with the Cu-centered 3d^10^ → 3d^9^4s^1^ transition (LMCT/d–s), whereas the low-energy band (295 nm) was attributed to a cluster-centered transition originating from d^10^–d^10^ overlap in a clustered structure with short Cu–Cu distances [113,114]. These assignments were inspired by model studies [115] on Cu_4_X_4_L_4_ complexes (X = Cl, Br, and I; L = pyridine or its derivative). It should be noted, however, that the cluster-centered transition in the model complexes was described in a different way—as a mixed d–s/XMCT with Cu–Cu interactions mediated by bonding *σ*-orbitals, delocalized over the Cu_4_ core, originating from Cu 4s orbitals [115]. As can be seen, the spectral properties of Cu-MTs, particularly the nature of the low-energy transition, have not yet been reliably explained, and it is tempting to speculate that some of the observed features can be ascribed to interactions of Cu(I) with disulfides present in the experimental system. CD studies on Cu-MTs are not conclusive in this respect, as both positive and negative Cotton effects at around 300 nm have been observed [113,114,116,117]. The Cu(I)–thiolate cluster in the DNA-binding domain of yeast ACE1 transcription factor shows no CD signal above 250 nm [118].

An additional argument favoring the importance of Cu(I) disulfides is the observation that the formed Cu(I) complexes (presumably t-IM1 and IM2) remain quite stable at the end of the autocatalytic phase of the studied autoxidation (Figure 7), when the Cu(I)–thiolates should be, as proposed in Scheme 1, oxidized to disulfides. This interpretation is supported by the findings of others that copper is partially found in its cuprous form even at the end of copper-catalyzed cysteine autoxidation [36,42].

## 4. Materials and Methods

The following chemicals were used as provided by the suppliers: *N*-acetyl-l-cysteine (>99%, Sigma-Aldrich, Darmstadt, Germany), ABTS diammonium salt (purum, >99%, Fluka Chemie GmbH, Buch, Switzerland), K_2_S_2_O_8_ (p.a., 99%, Merck, Darmstadt, Germany), HClO_4_ (p.a., 70–72%, Merck, Darmstadt, Germany), CuSO_4_·5H_2_O (p.a., Lachema, Brno, Czech Republic), and Cu(ClO_4_)_2_·6H_2_O (98%, Sigma-Aldrich, St. Louis, MO, USA).

Primary stock solutions of ABTSr were prepared as previously described [52] and stored frozen. The concentration of ABTSr in reaction solutions was determined spectrophotometrically. The stock solution of ABTSr contained also ABTS in an approximate ratio ABTSr/ABTS 2:1.

The reaction was performed in a quartz cell (1 cm optical path) in a reaction volume of 1.8 mL and directly followed by UV/Vis absorption and CD spectroscopies. The atmospheric oxygen was present in the capped cell in a sufficient superstoichiometric amount. Stirring of the reaction solution had a negligible effect on the reaction kinetics.

Absorption spectra were obtained on Agilent 8453 diode-array spectrophotometer in the spectral range of 190–1100 nm with a cycle time of 1, 3, or 20 s. CD spectra were recorded on a Jasco J-815 instrument (Tokyo, Japan) in the spectral range of 250–445 nm with a cycle time of 2 min (a scanning speed of 200 nm/min, time response 2 sec, data pitch 0.1 nm) and in 260–350 nm with a cycle time of 1 min (a scanning speed 100 nm/min, time response 1 s, data pitch 0.1 nm). After a baseline correction, the absorption spectra were expressed in terms of absorbance or differential absorbance (Δ*A*) in the case of difference absorption spectra (usually obtained by subtracting the spectra acquired at two discrete times). The baseline-corrected CD spectra were expressed as ellipticity (hereinafter referred to as CD signal) or differential ellipticity (ΔCD). The ellipticity time course was also recorded at a fixed wavelength of 305 nm with a data pitch of 10 s and the shutter on between records.

To reduce the exposure of the measured sample by UV light from a polychromatic source used by the diode-array device, the reaction kinetics were also followed by simultaneous recordings of absorbance at two discrete alternating wavelengths 300 and 400 nm on a Lambda 25 Perkin-Elmer UV/Vis spectrophotometer (cycle 17 s).

## 5. Conclusions and Perspectives

In summary, two intermediates, IM1 and IM2, have been detected in the reaction of NAC with Cu^2+^ ions at an equimolar ratio of the reactants in an aqueous acidic medium. IM1 shows an intense absorption band with a maximum at about 300 nm, while IM2 is characteristic of CD spectrum with two positive Cotton effects at 280 and 333 nm, and a strong negative one at 305 nm. The observed UV/Vis absorption and CD spectral features are attributed to a Cu(I) → S-S MLCT transition, providing that IM1 consists of two stereoisomers with a substantial cancellation of the CD signals. Both intermediates are capable of a rapid electron exchange with the ABTS free radical. The obtained spectral and kinetic data support the hypothesis that IM1 and IM2 are disulfide bridged dicopper(I) complexes that are likely to be the actual catalysts in the NAC autoxidation and ABTSr scavenging. This hypothesis seems to be in line with other reported observations regarding copper-catalyzed autoxidation of thiols, which have not been fully understood.

Although the studied reaction is too fast to trace the intermediates under neutral pH conditions, the proposed mechanisms deserve consideration of a possible relationship to biological processes. As the transient Cu(I) species, Cu(I)–thiolates, and possibly Cu(I) disulfides, show radical scavenging activity, an antioxidant functioning toward and O_2_^•−^ radicals could be expected. In contrast, when the level of ROS is decreased and the Cu(I) transients are sufficiently stable, their slow reactions with O_2_ may lead to the production of superoxide and hydrogen peroxide. This way, the Fenton process of ^•^OH radical formation could be initiated. In addition, or alternatively, Cu complexes capable of catalytic dioxygen activation could play an important role in the oxidation stress. On the other hand, such complexes as two-electron reductants may well be efficient scavengers of H_2_O_2_. Thus, both anti- and pro-oxidant behavior of thiols in the presence of copper ions should be considered. The resulting effect is probably complex and influenced by many factors determining the actual state of the biosystem. Such factors, for example, include the actual status in GSH/GSSG ratio (GSSG = oxidized GSH), the level of dioxygen, H_2_O_2_, ascorbate, and copper-binding proteins or other Cu ligands.

The presented results suggest that the interaction of Cu(I) with disulfides could be more significant than previously anticipated. Biological functions of various cysteine-rich proteins with the potential to form intra- or intermolecular disulfide bridges have been proposed to be associated with copper ions. For example, copper resistance protein CueP from *Salmonella enterica* binds Cu^+^ with very high affinity and has been recently shown to cooperate with a disulfide reductase system associated with increased copper tolerance of the pathogenic bacteria [119]. CueP is known as a metallochaperone delivering copper to Cu/Zn-superoxide dismutase, but a direct cell protective effect of CueP against a Cu^2+^/H_2_O_2_ treatment has also been demonstrated [120]. A possible role of disulfide bonds in amyloid fibril formation is currently discussed [121,122]. An interaction of Cu(I) of Sco2 protein with the disulfide bond of apo-COX II protein is probably the crucial step in the assembly of the Cu_A_ site of human cytochrome *c* oxidase [123]. Moreover, a protective effect of the disulfide bond against the areal oxidation of Cu(I) in copper-substituted rubredoxin has been suggested [44].

Special attention should be paid to GSH in search of the possible formation and biological activity of putative Cu^I^_2_GSSG and Cu^I^_2_(GSSG)_2_ complexes. Complexes of GSH with Cu(I) [64,124,125,126] and GSSG with Cu(II) [127,128,129,130,131] have been thoroughly studied, but there are still uncertainties about their structure under physiological conditions. However, almost no data are available on the possible presence of Cu(I)–GSSG complexes, although a possible role of such species in oxidation stress has been considered. Burkitt and Duncan [132] proposed a reaction of Cu(I)–GSSG with H_2_O_2_ generating ^•^OH radicals in the DNA–Cu(II)–H_2_O_2_–GSH system. Hrabarova et al. [133] observed that GSH loses its protective capacity against the hyaluronan degradation in a system with Cu(II) and ascorbate, when the molar ratio of GSH/Cu(II) approaches 1:1. In contrast, a pro-degradation effect was detected. Aliaga et al. [134] postulated a Cu(I)–GSSG intermediate during the reduction of Cu(II)–GSSG by GSH, supported by stoichiometric analysis and NMR studies. The appearance of species that are promising candidates for dicopper–disulfide complexes due to the intense spectral activity around 300 nm in the Cu(II)–GSH systems [37,43,64] makes such systems attractive for further research.

## Data Availability

Not applicable.

## Supplementary Materials

The following supporting information can be downloaded at: www.mdpi.com/article/10.3390/ijms23116199/s1.
