# Supplementary material for: Reaction of N-Acetylcysteine with Cu2+: Appearance of Intermediates with High Free Radical Scavenging Activity: Implications for Anti-/Pro-Oxidant Properties of Thiols
Source: Int J Mol Sci. 2022 May 31;23(11):6199. doi: 10.3390/ijms23116199 (PMC9181168; doi:10.3390/ijms23116199)
Supplement: Supplementary file 1 [file ijms-23-06199-s001.zip › ijms-1734599-supplementary.pdf]

Supporting Information for

Reaction of *N*-acetylcysteine with Cu^{2+}: Appearance of intermediates with high free radical scavenging activity. Implications for anti-/pro-oxidant properties of thiols

Ivan Valent[1,]*, Lucie Bednárová[2], Igor Schreiber[3,4], Juraj Bujdák[1], Katarína Valachová[5], Ladislav Šoltés[5]

[1] Department of Physical and Theoretical Chemistry, Faculty of Natural Sciences, Comenius University, Mlynská dolina, Ilkovičova 6, 842 15 Bratislava, Slovakia

[2] Institute of Organic Chemistry and Biochemistry of the Czech Academy of Sciences, Flemingovo náměstí 542/2, 160 00 Praha 6, Czech Republic

[3] Department of Chemical Engineering, Faculty of Chemical Engineering, University of Chemistry and Technology, Prague, Technická 5, 166 28 Praha 6, Czech Republic

[4] Global Change Research Institute, Czech Academy of Sciences, Bělidla 986/4a, 603 00 Brno, Czech Republic

[5] Centre of Experimental Medicine of Slovak Academy of Sciences, Dúbravská cesta 9, 841 04 Bratislava, Slovak Republic

E-mail: ivan.valent@uniba.sk

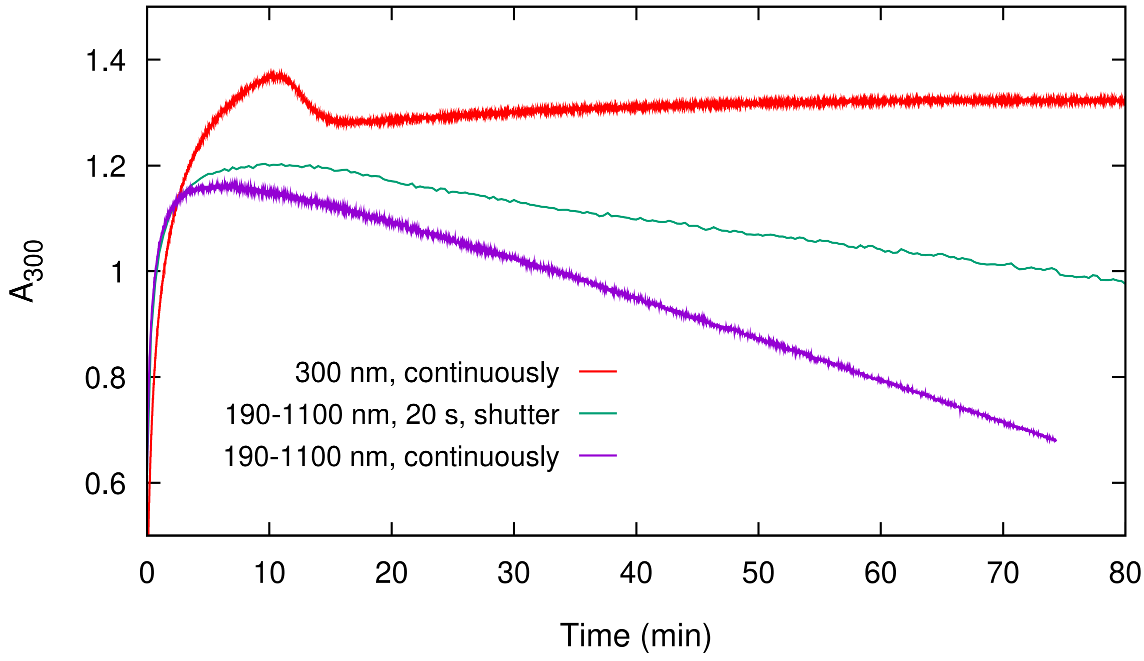

Figure S1. Effect of illumination regime on time dependence of the absorbance at 300 nm during various spectrophotometric recordings. **Red line:** continuous monochromatic illumination (λ = 300 nm, Lambda 25 Perkin-Elmer UV/Vis spectrophotometer); **Green line:** short (<1 s) periodic polychromatic flashes (λ = 190 – 1100 nm, interval 20 s, Agilent 8453 diode-array spectrophotometer with shutter); **Magenta line:** continuous polychromatic illumination (λ = 190 – 1100 nm, Agilent 8453 diode-array spectrophotometer). Reaction conditions: 300 µM Cu^{2+}, 300 µM NAC, 10 mM $HClO_4$, 20 °C.

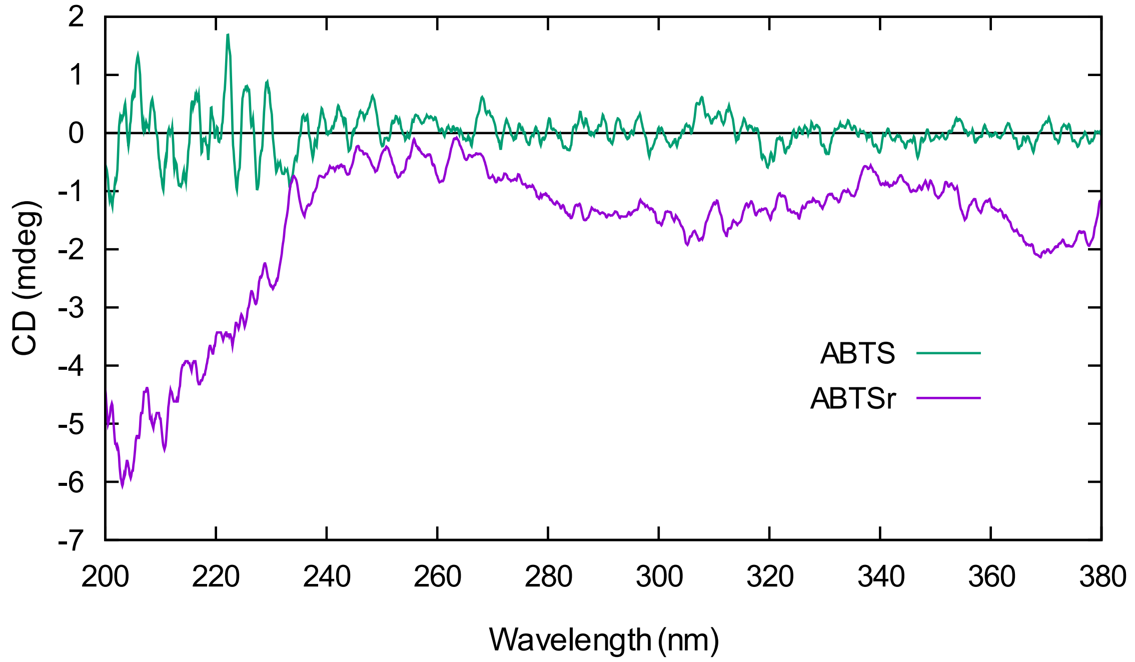

Figure S2. CD spectra of ABTS and ABTSr. **Green line:** CD spectrum of a solution containing ~30 µM 2,2′-azino-bis(3-ethylbenzothiazo-line-6-sulfonic acid) diammonium salt (ABTS) and 40 mM $HClO_4$. Scan rate 100 nm min^{-1}, high sensitivity, 20 ℃. **Magenta line:** CD spectrum of a solution containing ~24 µM ABTS radical (ABTSr), ~12 µM ABTS, 360 µM $CuSO_4$ and 48 mM $HClO_4$. The spectrum represents an average of four scans at the scan rate of 100 nm min^{-1}.

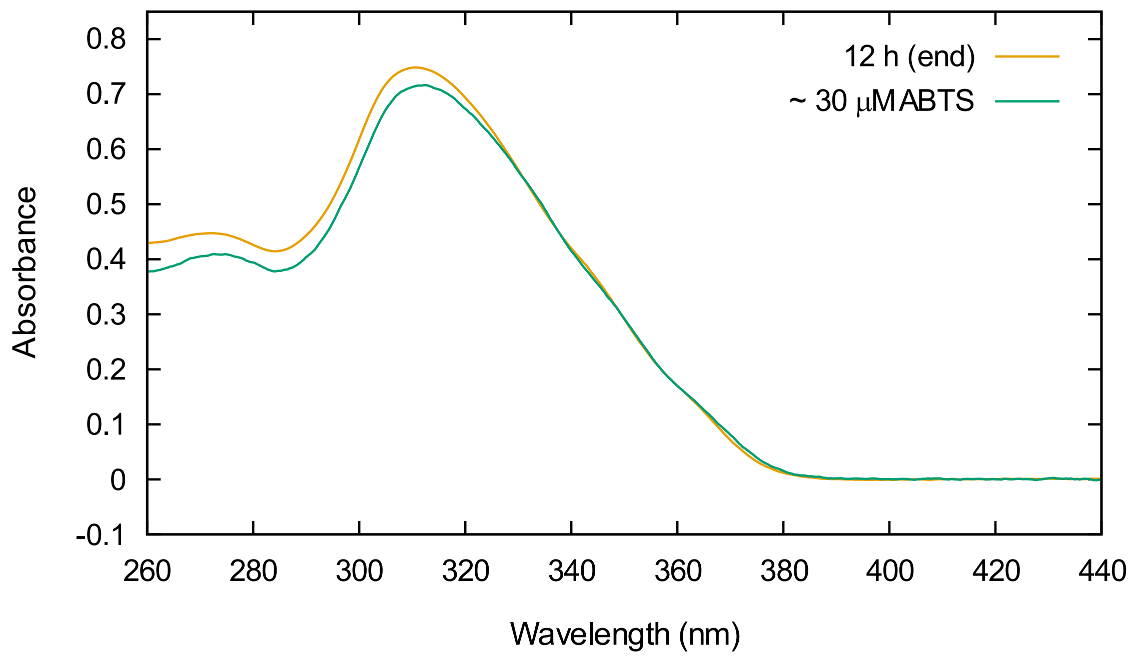

Figure S3. Comparison of the ABTS absorption spectrum from the reaction with a reference. **Yellow line:** absorption spectrum after 12 hours (end) from the beginning of the reaction (see Figure 8b in the main text). **Green line:** absorption spectrum of ~30 µM ABTS in 40 mM $HClO_4$.

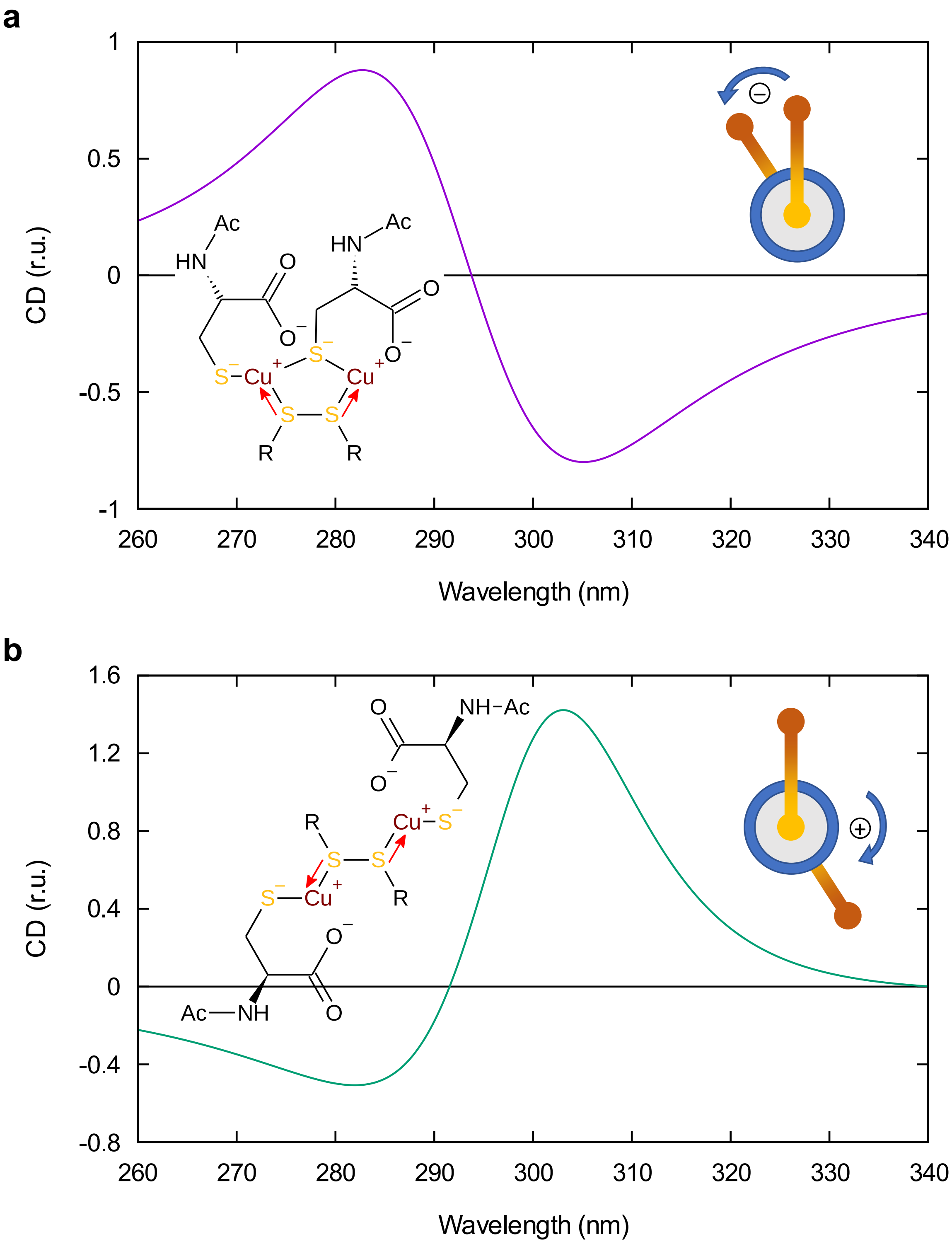
a
CD (r.u.)
1
0.5
0
-0.5
-1
260
270
280
290
300
310
320
330
340
Wavelength (nm)
Ac
HN
O
O⁻
S⁻
Cu⁺
S
S
R
R
b
CD (r.u.)
1.6
1.2
0.8
0.4
0
-0.4
-0.8
NH-Ac
Ac—NH
Wavelength (nm)

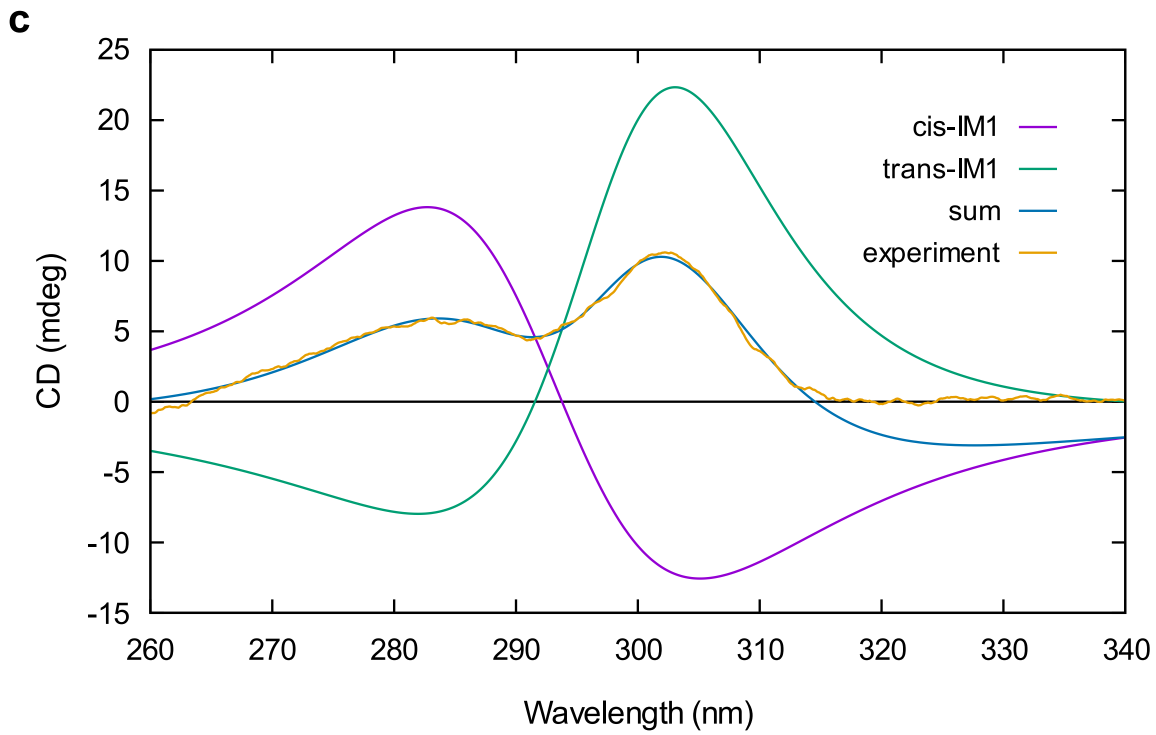

Figure S4. An illustrative suggestion for the exciton chirality of the IM1 isomers. (**a**, **b**) Negative (a)/ positive (b) exciton chirality of *cis*/*trans*-IM1. The vectors of transition dipole moment (red arrows) constitute a counterclockwise (a) and a clockwise (b) screw, which determines the signs of the corresponding Cotton effects of an exciton split: negative first (at longer wavelength) and positive second (at shorter wavelength) Cotton effects for *cis*-IM1 (a), and *vice versa* for *trans*-IM1 (b). As the actual conformation of the isomers is unknown, an opposite assignment of the exciton chirality for the IM1 isomers (positive for *cis*-IM1 and negative for *trans*-IM1) can also be suggested. However, the proposed chirality of *cis*-IM1 agrees with a similar exciton coupling in the IM2 molecule (see Figure S4a). The Cotton effects (in relative units) are simulated by normalized Lorentzian functions. (**c**) An appropriately scaled sum of the exciton-coupled Cotton effects of both IM1 isomers compared with an experiment. The shown experimental data (see Figure 5 for 80 min in the main article) represent the measured values subtracted with a background value of 4 mdeg.

a

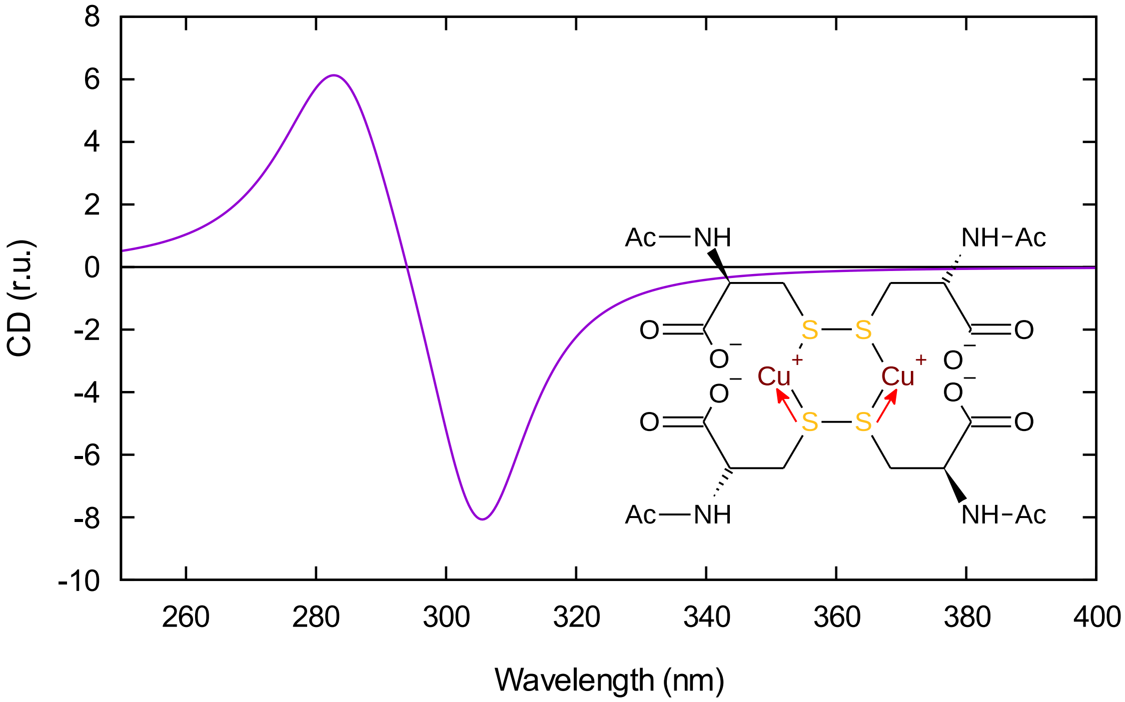

8
6
4
2
0
-2
-4
-6
-8
-10
CD (r.u.)
260
280
300
320
340
360
380
400
Wavelength (nm)
Ac—NH
NH−Ac
O
O
O−
O−
S
S
Cu+
Cu+
O−
O−
O
O
S
S
Ac—NH
NH−Ac

b

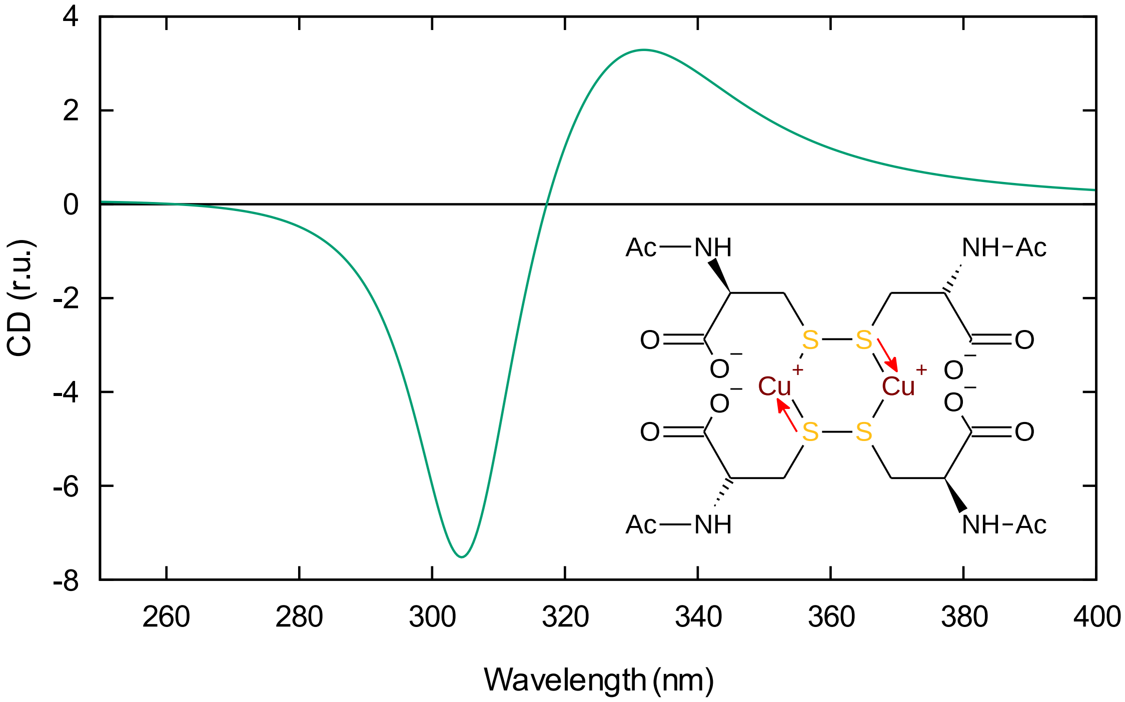

4
2
0
-2
-4
-6
-8
CD (r.u.)
260
280
300
320
340
360
380
400
Wavelength (nm)
Ac—NH
NH−Ac
O
O
O−
O−
S
S
Cu+
Cu+
O−
O−
O
O
S
S
Ac—NH
NH−Ac

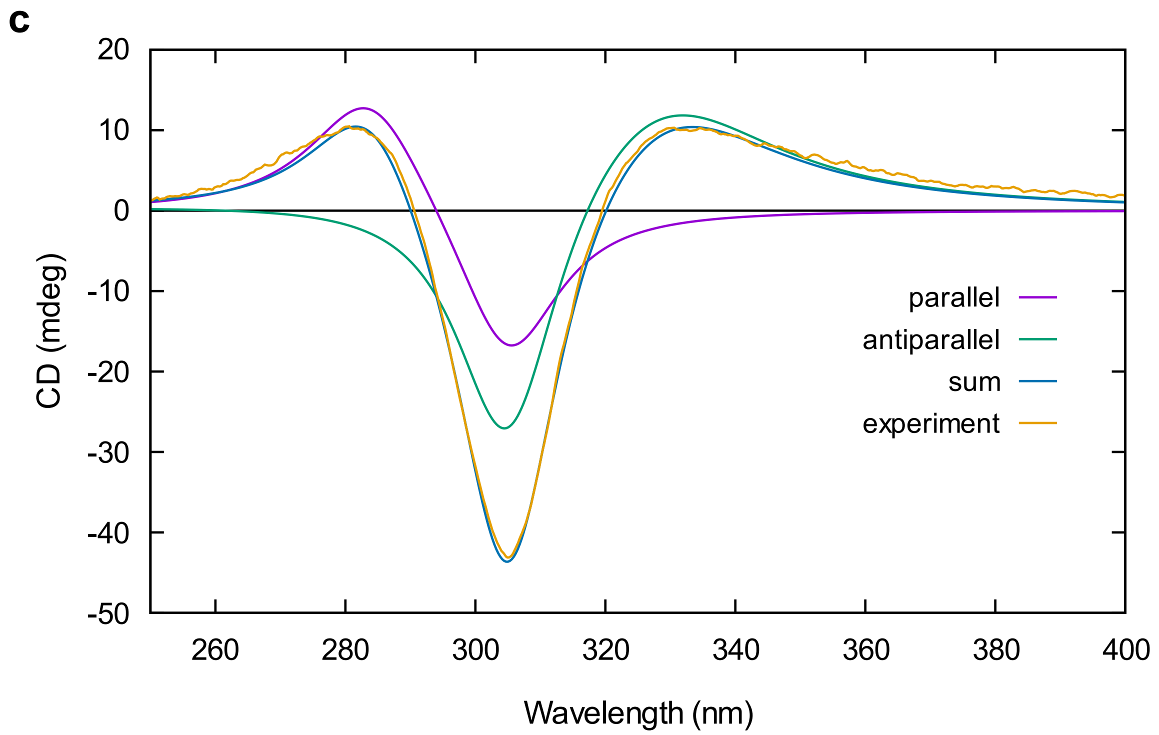

Figure S5. A possible interpretation of the CD spectrum of IM2. (**a**) Negative exciton chirality resulting from a parallel coupling of the transition dipole moments (red arrows) with a positive interaction energy. The exciton split compares very well with the similar coupling in *cis*-IM1 (Figure S3a). (**b**) Positive exciton chirality resulting from an antiparallel coupling of the transition dipole moments with a negative interaction energy. The Cotton effects (in relative units) are simulated by normalized Lorentzian functions. (**c**) A sum of the appropriately scaled exciton-coupled Cotton effects of both components compared with an experiment. The shown experimental data represent a time difference CD spectrum (Δt = 50 min minus 38 min) from a standard experiment (300 µM Cu^{2+}, 300 µM NAC, 40 mM $HClO_4$, 20 °C). A sum of the rotational strengths (R) of the three observed Cotton effects, calculated as integrals $\int CD(\lambda)/\lambda d\lambda$ along the x-axis (*i.e.*, wavelength, λ), is very close to zero ($|R_+/R_-| \approx 1.02$) in agreement with the suggested interpretation of the spectrum.
